# Poly (γ) glutamic acid: a unique microbial biopolymer with diverse commercial applicability

**DOI:** 10.3389/fmicb.2024.1348411

**Published:** 2024-02-13

**Authors:** Khaled Elbanna, Fatimah S. Alsulami, Leena A. Neyaz, Hussein H. Abulreesh

**Affiliations:** ^1^Department of Biology, Faculty of Science, Umm Al-Qura University, Makkah, Saudi Arabia; ^2^Research Laboratories Unit, Faculty of Science, Umm Al-Qura University, Makkah, Saudi Arabia; ^3^Department of Agricultural Microbiology, Faculty of Agriculture, Fayoum University, Fayoum, Egypt

**Keywords:** poly (γ-glutamic acid), (γ-PGA), microbial biopolymer, medical applications, food applications, pharmaceutical applications

## Abstract

Microbial biopolymers have emerged as promising solutions for environmental pollution-related human health issues. Poly-γ-glutamic acid (γ-PGA), a natural anionic polymeric compound, is composed of highly viscous homo-polyamide of D and L-glutamic acid units. The extracellular water solubility of PGA biopolymer facilitates its complete biodegradation and makes it safe for humans. The unique properties have enabled its applications in healthcare, pharmaceuticals, water treatment, foods, and other domains. It is applied as a thickener, taste-masking agent, stabilizer, texture modifier, moisturizer, bitterness-reducing agent, probiotics cryoprotectant, and protein crystallization agent in food industries. γ-PGA is employed as a biological adhesive, drug carrier, and non-viral vector for safe gene delivery in tissue engineering, pharmaceuticals, and medicine. It is also used as a moisturizer to improve the quality of hair care and skincare cosmetic products. In agriculture, it serves as an ideal stabilizer, environment-friendly fertilizer synergist, plant-growth promoter, metal biosorbent in soil washing, and animal feed additive to reduce body fat and enhance egg-shell strength.

## 1 Introduction

**Figure 1:**
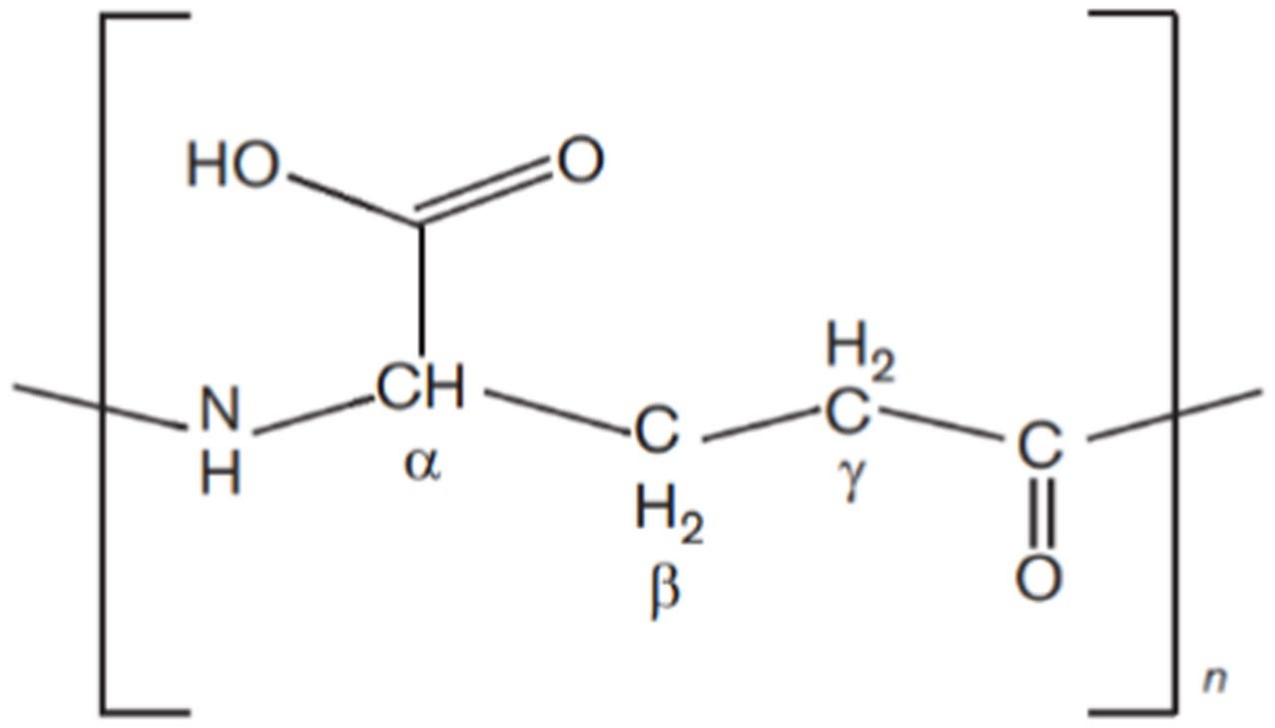
Chemical structure of γ-PGA. D- and L-glutamic acid units are joined together via amide linkages between α-amino and γ-carboxylic acid groups.

Microbial biopolymers are gaining global popularity due to their eco-friendly, degradable, and non-toxic nature as compared to synthetic non-degradable polymers. Biopolymers are in high demand but low-yielding microbial strains and high production costs restrict their commercialization (Kreyenschulte et al., 2014). Polygamma glutamic acid (γ-PGA) is an expensive biopolymer and a few milligrams cost several dollars. It is a unique anionic homopolyamide of D- and L-glutamic acid units, which are joined together via amide linkages between α-amino and γ-carboxylic acid groups (Shih et al., 2001; Li D. et al., 2022; Li S et al., 2022) (see Figure 1).

γ-PGA is a completely biodegradable extracellular product that is water-soluble, edible, non-immunogenic, and harmless to humans (Young and Richard, 1988; Yoon et al., 2000). The unique properties of γ-PGA favor its applications in pharmaceuticals, medicine, cosmetics, healthcare, agricultural production, foods, and water treatment (Da Silva et al., 2014). Currently, *Bacillus* species are used for the safe and natural microbial synthesis of γ-PGA. The features such as molecular weight, molecular composition, synthesis productivity, and yield determine the biological properties and industrial application of γ-PGA. Enhanced industrial demand highlights the importance of biopolymers such as γ-PGA, which could affect the existing industries and traditional commercial polymers. However, the low yield and higher costs of γ-PGA production hinder its industrial applications. These limitations can be overcome by improving the γ-PGA biosynthesis process through genetic manipulation, screening of more potent microbial producers, the use of a better culture medium, and the optimization of microbial culturing conditions. The following sections emphasize the novel theoretical studies and advanced techniques of γ-PGA microbial synthesis in the future (Bajaj et al., 2011; Da Silva et al., 2014; Li et al., 2022).

The reasons for microbial γ-PGA production remains not fully understood; however, it is suggested that it is to protect the cells from phages, antibodies, antimicrobial peptides, and detrimental environmental conditions. Moreover, it facilitates microbial adhesion to nutrient particles and serves as a nitrogen and carbon source during starvation (Mesnage et al., 1998; Luo et al., 2016; Sirisansaneeyakul et al., 2017). γ-PGA function depends on the environment, producing microorganisms, and released or bound peptidoglycan. Peptidoglycan-bound γ-PGA could confer virulence or serve as a glutamate source under starving conditions (Kimura et al., 2004; Kocianova et al., 2005) whereas environmental release of γ-PGA helps in an organism’s survival under unfavorable conditions (McLean et al., 1990).

The capsules of virulent *B. anthracis* strains solely contain γ-D-PGA where the D enantiomer contributes to non-immunogenic properties (Tomcsik and Szongott, 1933; Zwartouw and Smith, 1956; Candela and Fouet, 2006). Contrarily, some soil bacterial species are known for the environmental release of γ-PGA that helps in toxic metal ion sequestration for enhanced resistance under unfavorable environments (McLean et al., 1990). *Natrialba aegyptiaca*, *Planococcus halophilus,* and *Sporosarcina halophila* employ γ-PGA to mitigate higher concentrations of local salt for better survivability under hostile environments (Kandler et al., 1983; Hezayen et al., 2001). Marine eukaryotic *Cnidaria* explosively uses stinging cells (nematocysts) for locomotion, protection, and the capturing of prey. They produce large amounts of γ-PGA that assist in triggering the cellular explosive reaction (Weber, 1990). γ-PGA has been reported in mice neurons, which alters tubulin and Ca^2+^ interaction and tubulin-associated proteins to regulate the microtubule dynamics (Edde et al., 1990). *Bacillus amyloliquefaciens* C06 uses γ-PGA for enhanced motility and biofilm formation. γ-PGA sticks the cells together in a coordinated pattern for better absorption of essential environmental nutrients to enhance microbial motility (Liu et al., 2010).

## 2 PGA producing microorganisms

Ivannovics and Bruckner (1937) discovered the PGA as a *Bacillus anthracis* capsule that was released into the medium on aging, cell autolysis, and autoclaving (Ashiuchi and Misono, 2005). Several γ-PGA-producing strains have been identified but *Bacillus* species, particularly *B. subtilis* and *B. licheniformis*, remain the most potent γ-PGA producers (Luo et al., 2016; Sirisansaneeyakul et al., 2017). Shih et al. (2001) have categorized γ-PGA-producing bacteria into two groups based on their nutrient requirements for γ-PGA synthesis. One group needs L-glutamic acid in the growth medium whereas the other group does not require L-glutamic acid for γ-PGA production. L-glutamic acid-dependent bacteria include *B. subtilis* CGMCC 0833, *B. licheniformis* 9945a (Wu et al., 2010b), *B. subtilis* (chungkookjang) (Ashiuchi et al., 2003a), *B. subtilis* (natto) ATCC 15245 (Bhat et al., 2013), and *B. licheniformis* NK-03 (Cao et al., 2010). γ-PGA producers that do not require L-glutamic acid include *B. subtilis* C10 (Zhang H. et al., 2012), *B. subtilis* C1 (Shih et al., 2005), and *B. amyloliquefaciens* LL3 (Cao et al., 2011). The PGA yield of L-glutamic acid-dependent bacteria increases with the rise in medium concentration of L-glutamic acid. However, these bacteria can also adopt the *de novo* pathway for γ-PGA production in the absence of exogenous L-glutamic acid (Kunioka and Goto, 1994; Buescher and Margaritis, 2007). A simple fermentation process and low-cost L-glutamate-independent γ-PGA producers are more desirable for industrial production as compared to glutamate-dependent γ-PGA producers (Cao et al., 2011). However, their lower γ -PGA productivity than L-glutamate-dependent producers hinders industrial application. Therefore, genetically engineered non-glutamate-dependent producers such as *B. amyloliquefaciens* NK-1 (Feng et al., 2014) and laboratory strains such as *B. subtilis* 168, *E. coli*, and *B. subtilis* MA41 have been developed for a higher γ-PGA yield (Ashiuchi et al., 2006; Cao et al., 2013; Scoffone et al., 2013).

### 2.1 γ-PGA production with *Bacillus licheniformis*

*Bacillus licheniformis* is widely used for γ-PGA production; particularly, the *B. licheniformis* 9945a (NCIM 2324) strain is well known for γ-PGA production. Multiple studies have optimized γ-PGA production to retrieve maximum yield. Bajaj et al. (2009) adopted solid-state fermentation to enhance *B. licheniformis* NCIM 2324-based γ-PGA production. They followed the ‘one factor at a time’ method to examine the effects of moisture content, solid substrates, pH, nitrogen and carbon sources, TCA cycle intermediates, and amino acids on γ-PGA production. The optimized media produced a maximum γ-PGA yield of 98.64 mg/g dry solids with solid fermentation. Response surface methodology was further applied to optimize the nutrient concentrations in the medium, which were experimentally tested. A significantly higher γ-PGA yield (26.12 g/L) was noted with the optimized medium [glycerol (62.4 g/L); ammonium sulphate (8.0 g/L); citric acid (15.2 g/L); and L-glutamic acid (20 g/L)] as compared to the basal medium (5.27 g/L). The produced γ-PGA had a molecular mass of 2.16105 Da. Based on these findings, the authors developed a more efficient system and obtained a γ-PGA production of 35.75 g/L using the *B. licheniformis* NCIM 2324 strain. This is the highest reported production of γ-PGA with a *B. licheniformis* strain in the submerged fermentation process. This was achieved by supplementing *B. licheniformis* NCIM 2324 medium with L-glutamine (0.07 g/L) and α-ketoglutaric acid (1.46 g/L), which served as metabolic precursors for γ-PGA production. γ-PGA yield was considerably high (35.75 g/L) in the metabolic precursor-supplemented medium in comparison to the medium without these precursors (26.12 g/L). Thus, precursors facilitated better utilization of L-glutamic acid by the studied strain. Mabrouk et al. (2012) followed the Plackett–Burman design to optimize the medium [glucose (50 g/L); K*_2_* HPO_4_ (6.4 g/L); NH_4_Cl (3 g/L); MgSO_4_.7H_2_O, (0.8 g/L); yeast extract (2 g/L); NaCl (0.8 g/L); FeSO_4_.4H_2_O (0.006 g/L); CaCl_2_.2H_2_O (0.00084 g/L); trace element solution (0.1 mL), and culture volume (25 mL)] and achieved a γ-PGA yield of 28.2 g/L using an exogenous glutamate-independent strain *B. licheniformis* A13. Medium volume and yeast extract mainly affected the γ-PGA production. The Plackett–Burman experimental design has also been applied to assess the culture requirements of *B. licheniformis* SAB26 (Soliman et al., 2005). γ-PGA production was evaluated against fifteen variables mainly including (NH_4_)_2_SO_4_, K_2_HPO_4_, KH_2_PO_4_, and casein hydrolysate. The use of an L-glutamic acid nitrogen source alleviated the γ-PGA production of *B. licheniformis* SAB-26 and thus it was classified as a glutamate-independent γ-PGA producing strain. Ogunleye et al. (2015) noted a three times higher γ-PGA yield (33.5 g/L) on an optimized medium as compared to a basal medium.

### 2.2 γ-PGA production with *Bacillus subtilis*

Bovarnick (1942) discovered that *B. subtilis* fermentation leads to γ-PGA secretion into the medium. This discovery attracted the researchers to further investigate *B. subtilis*-based γ-PGA production. *B. subtilis* strains have been more thoroughly studied for γ-PGA production than *B. licheniformis* strains. Scoffone et al. (2013) recently evaluated the γ-PGA production by knocking out two γ-PGA-degrading enzyme genes (*ggt* and *pgdS*) in *B. subtilis* 168 (laboratory strain). They studied the effects of double (deletion of both genes) and single (one gene at a time) mutations on γ-PGA yield. Single mutations did not significantly improve the γ-PGA production whereas double mutation led to a twofold increase (40 g/L) in γ-PGA yield compared to the WT strain. However, the weight average molecular mass and number average molecular mass of produced γ-PGA were comparatively lower in double-mutant strains than in single-mutant and WT strains. The highest molecular mass (36,106 Da) was noted in the *pgdS* mutant strain, which could be attributed to decreased endo-degradation activities. Huang et al. (2011) have reported cost-effective high-yield and large-scale γ-PGA production with *B. subtilis* ZJU-7 (*B. subtilis* CGMCC1250). They stated that 30 g/L L-glutamate, 40 g/L yeast extract, 20 g/L initial glucose, and a glucose concentration range of 3–10 g/L significantly enhanced the γ-PGA production (1.4 to 3.2-fold) following a fed-batch approach in comparison to batch fermentation. Overall, γ-PGA concentration and productivity remained at 101.1 g/L and 2.19 g/L, respectively. Jian et al. (2005) optimized the solid-state fermentation of *B. subtilis* CCTCC202048 for γ-PGA production. They achieved a maximum γ-PGA yield of 83.61 g/L (kg dry solids) with a mixed substrate (11:9 w/w) of soybean cake powder and wheat bran along with glutamate (40.14 kg/L), NH_4_NO_3_ (20.05 kg/L), citric acid (18.50 kg/L), and mineral salts (FeCl_3_.6H_2_O, MgSO_4_.7H_2_O, MnSO_4_.H_2_O, and CaCl_2_.2H_2_O). Low production costs and high yield favor solid-state fermentation for large-scale γ-PGA production. Shih et al. (2005) used the *B. subtilis* C1 strain to synthesize a novel glycerol-γ-PGA derivative in an L-glutamate-lacking medium. *B. subtilis* C1 depended on glycerol and citric acid for γ-PGA production and the absence of any of these compounds significantly impacted the yield. The conjugate’s molecular mass (16,107 Da) was found to be higher than the super-high-molecular mass of γ-PGA reported by Park et al. (2005), which could be attributed to the glycerol in the medium. The conjugate presented an γ-PGA to glycerol ratio of 10:1 and had a higher D-glutamic acid units (97%) concentration as compared to L-glutamic acid units. Interestingly, the enantiomeric composition of glycerol-γ-PGA conjugate remained unaffected against Mn^2+^. Contrarily, Wu et al. (2006) have established the effects of Mn^2+^ on the enantiomeric and stereochemical composition of *B. subtilis* NX-2 produced γ-PGA. The D-glutamate proportion was noted to rise (18 to 77%) in response to variations in Mn^2+^ concentrations (0–0.09 g/L). Mn^2+^ affected γ-PGA stereochemical properties by modifying glutamate racemase activity (Ogunleye et al., 2015).

### 2.3 γ-PGA production with *Bacillus amyloliquefaciens*

Cao et al. (2013) cloned γ-PGA synthase genes (*racE* and *pgsBCA*) from a non-L-glutamate-dependent γ-PGA producer *B amyloliquefaciens* LL3 and an L-glutamate-dependent γ-PGA producer *B. licheniformis* NK03 in *E. coli* JM109 and evaluated γ-PGA yield. *pgsC* and *pgsB* genes of both strains shared high similarities of 93.96 and 93.13% whereas *racE* and *pgsA* genes presented 84.5 and 78.53% similarities, respectively. The engineered strains (4) yielded γ-PGA in both L-glutamate and glucose media after 24 h of culturing. Irrespective of the harboring vector, *PgsBCA* of *B. amyloliquefaciens* LL3 exhibited better catalytic efficiency than *B. licheniformis* NK-03, and *B. amyloliquefaciens* LL3-pgsBCA yielded a higher γ-PGA quantity than *B. licheniformis* NK-03-pgsBCA. *B. amyloliquefaciens* LL3-derived *RacE* and *PgsBCA* and *B. licheniformis* NK-03-derived *RacE* and *PgsBCA* displayed significantly enhanced D-isomer content and productivity of γ-PGA in comparison to *B. amyloliquefaciens* LL3-derived *PgsBCA* and *B. licheniformis* NK-03-derived *PgsBCA*. It depicts that *racE* incorporation enhanced the γ-PGA productivity and D-isomer content. *B. subtilis* and *Corynebacterium glutamicum* were co-cultured using a mixed carbon source (sucrose and glucose) to avoid the addition of exogenous L-glutamic acid, which reduced the production cost and fermentation time, and γ-PGA had an average molecular mass of 1.246106 Da (Jian et al., 2005).

### 2.4 γ-PGA production with other *Bacillus* species

*Bacillus anthracis*-based production of pure γ-PGA D-enantiomer has been established (Zwartouw and Smith, 1956). However, the production mechanism of *B. anthracis* differs from other *Bacillus* species as its γ-PGA remains bound to peptidoglycan and is not secreted into the medium. Thus, it complicates the purification and recovery process, which involves cell autolysis and autoclaving. Moreover, *B. anthracis* toxicity also restricts industrial scale γ-PGA production. The anchored γ-PGA is associated with a non-immunogenic *B. anthracis* capsule, which is linked to the lethal toxin (Ezzell et al., 2009). Therefore, the γ-PGA-anchoring capping gene should be targeted to avoid *B. anthracis* toxicity (Candela and Fouet, 2006). *Bacillus thuringiensis* vs. Monterrey strain BGSC 4AJ1 is also known to produce γ-PGA capsules like *B. anthracis* (Cachat et al., 2008). *B. anthracis* (Ames) and *B. thuringiensis* sv. Monterrey strain BGSC 4AJ1 share four alleles (gmk-1, tpi-1, pta-1, and pur-1) while differing in three other alleles (pycA-52, glpF-57, and ilvd-52) by three, two, and two nucleotides, respectively. γ -D-PGA-producing plasmid (pAJ1-1) genes share similarities with *B. anthracis*. The discovery of γ-PGA capsule in the *B. thuringiensis* strain indicates its potential pathogenicity under specific conditions. Poli et al. (2015) isolated *Bacillus horneckiae* strain APA from a shallow hydrothermal vent of Panarea Island, Italy. They characterized it as an extracellular poly-γ-glutamic acid (γ-PGA)-producing strain and studied its immunomodulatory and antiviral effects against the HSV-2 virus (Herpes simplex virus type 2). Tarui et al. (2005) adopted the *Agrobacterium* infection technique to introduce indispensable *pgsBCA* complex into tobacco leaves. γ-PGA production was noted only in plant tissue with all three *pgsBCA* genes, which yielded 600 mg γ-PGA/g leaf material (Ogunleye et al., 2015).

### 2.5 Halophiles-based production of γ-PGA

The cell wall of Archaebacterium *Natronococcus occultus* is known to contain L-glutamate (Niemetz et al., 1997). *Natrialba aegyptiaca* strain 40 T is extremely halophilic and was the first archaebacterium to produce extracellular poly-γ-D-(glutamate) (PGA) (Hezayen et al., 2000, 2001). Then, archaebacterial strain 56 T was also characterized to produce exopolymer containing PGA (65% w/w), carbohydrates (15% w/w), and unidentified material (20% w/w) (Hezayen et al., 2002).

### 2.6 γ-PGA production from genetically modified microorganisms

Spizizen (1958) devised techniques to develop recombinant *Bacillus* strains for the commercial production of the *B. subtilis* enzyme. The transformation proved a significant milestone in molecular biology. Engineered plasmids are obtained either from natural plasmids or plasmids of closely related organisms, which are inducted into a host strain through electroporation, competent cell transformation, and protoplast fusion. Plasmids can be inherited, replicated, or transcribed into host strains. Recombination occurs over several generations during the fermentation process of *Bacillus* species. The deletion of plasmid sequences could lead to problematic replication and segregation steps in modified plasmids. Thus, the development of mutant strains lacking relevant recombination enzymes dramatically enhanced the stability of recombinant strains. Native promoters (α-amylase gene) directing the production of extracellular proteins (20 g/L) are frequently employed in industrial microbiology. Direct DNA insertion into chromosomes by designing constructs flanked with homologous sequences to chromosomal genes produce more stable clones. The knowledge of *Bacillus* species nucleotide sequence offers the possibility of novel modifications to produce strains with desired traits. Gene cloning via the encoding of novel plasmid enzymes into antibiotic-resistant *B. subtilis* hosts helps to maintain plasmid. The induction of a dal-bearing plasmid into a dal-mutant is performed into a dal-mutant, which is unable to produce D, L-alanine racemase. D, L-racemase is necessary for the production of the cell’s D-alanine component. The genetic constructs retain selective pressure on plasmid maintenance in these hosts. Chromosomal integration of novel enzyme-expressing genes could also generate high-yielding *B. subtilis* strains. The amplification of genes could facilitate the development of highly efficient enzyme-producing strains for large-scale fermentation (Gupta et al., 2002).

*B. licheniformis* strains are crucial for commercial applications as their enzyme-yielding efficiency is higher than *B. subtilis*. A high enzyme production in these strains needs a combination of high-performance expression systems from traditional high-yielding strains and multiple copies of the target gene. The ideal strains should have a genetically stable and chromosomally integrated gene with higher expression levels and product secretion. *B. licheniformis* and *B. subtilis* have successfully fulfilled these criteria (Markcus et al., 2004).

## 3 Mechanism of γ-PGA biosynthesis

**Figure 2:**
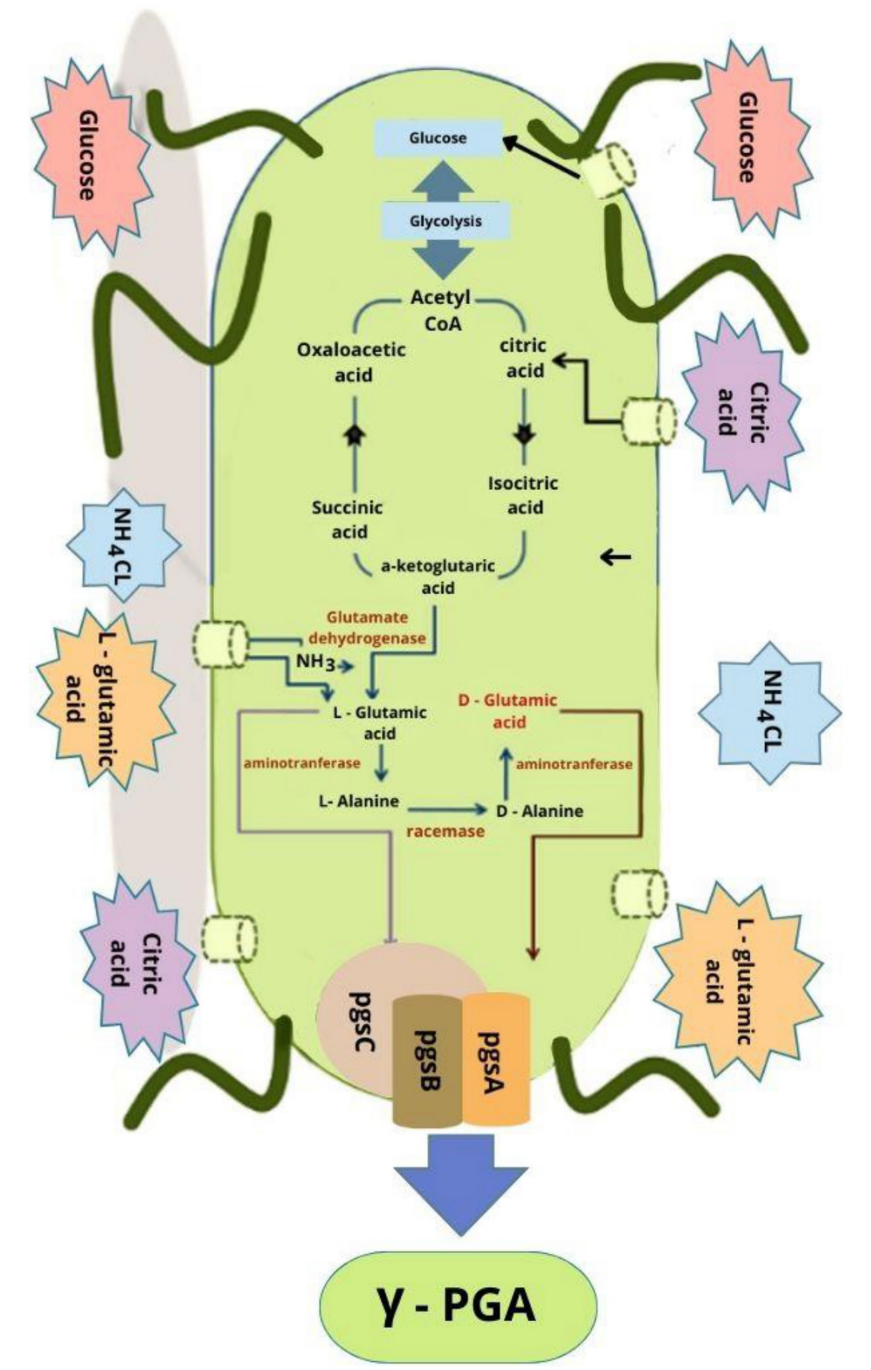
A proposed pathway of γ-PGA synthesis (Shih et al., 2001; Sirisansaneeyakul et al., 2017).

γ-PGA production is more expensive than traditional polymers. An understanding of the mechanism and γ-PGA biosynthesis-impacting metabolic gene clusters might help in developing improved strains for higher γ-PGA production (Ogunleye et al., 2015). Figure 2 presents the metabolic pathway of γ-PGA production and a related gene cluster. The prerequisite (L-glutamic acid) of γ-PGA production could be endogenous or exogenous. Endogenous availability activates the L-glutamic acid pathway that converts the carbon source in the medium into acetyl CoA via glycolysis followed by Krebs cycle-based synthesis of α-ketoglutaric acid. Two distinct pathways can convert α-ketoglutaric acid precursor into L-glutamic acid. Glutamate dehydrogenase converts α-ketoglutaric acid and ammonium chloride into L-glutamic acid in the absence of glutamine whereas 2-oxoglutarate aminotransferase catalyzes α-ketoglutaric acid and glutamine conversion to L-glutamic acid in the presence of L-glutamine. γ-PGA biosynthesis involves the activity of various enzymes such as peptidase, racemase, and synthase (Ashiuchi, 2010; Wang et al., 2017).

### 3.1 Racemization of γ-PGA

γ-PGA can be a homopolymer (poly -γ-D- glutamic acid, poly-γ-L-glutamic acid) or a heteropolymer (poly-γ-DL-glutamic acid). The growing peptide chain either incorporates L-glutamic acid monomers from the medium or is synthesized by enzymes (2-oxoglutarate aminotransferase or glutamate dehydrogenase) (Ashiuchi et al., 2001). However, racemase activity is necessary for D-glutamic acid synthesis, which catalyzes D-glutamic acid formation from L-glutamic acid and the process is referred to as racemization (Ashiuchi et al., 1998; Luo et al., 2016). *B. subtilis* contains two glutamate racemase homologous genes (*yrpC* and *racE* (*glr*)) (Ogunleye et al., 2015). These genes do not directly participate in γ-PGA biosynthesis but are essential for the strain’s growth in nutrient-rich (*racE*) and minimal medium (*yrpC*) (Wang et al., 2016). Contrarily, *glr* plays a key role in γ-PGA synthesis and forms D-glutamic acid from L-glutamic acid. The overexpression of the *glr* gene could enhance the D-glutamate enantiomeric ratio in *B. licheniformis* (Jiang et al., 2011).

### 3.2 γ-PGA synthesis and polymerization

**Figure 3:**
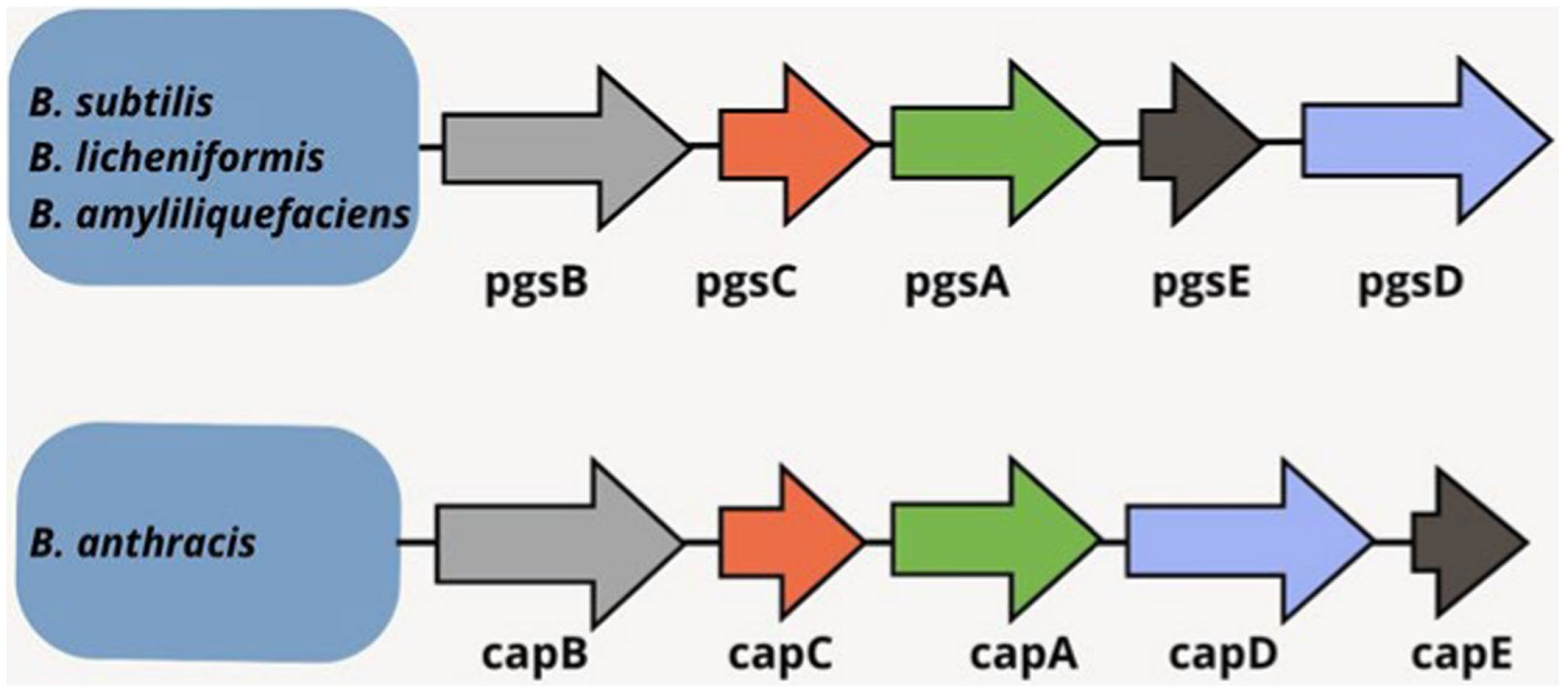
γ-PGA synthetase genes in *Bacillu*s spp. (Shih and Van, 2001; Candela and Fouet, 2006; Sirisansaneeyakul et al., 2017).

*B. anthracis* contains plasmid-encoded γ-PGA biosynthesis genes whereas such genes are chromosomally inherited in some *Bacillus* species (Ashiuchi and Misono, 2002; Ogunleye et al., 2015). The release or anchoring of synthesized γ-PGA depends on the gene function. Cap genes are crucial in *B. anthracis* for capsule formation where γ-PGA is attached to the cell surface. Contrarily, *B. licheniformis* or *B. subtilis* release γ-PGA outside the cells under the influence of *pgs* genes. *B. anthracis* cap genes (cap B, C, A, E) are homologous to *B. licheniformis* and *B. subtilis* cap genes (pgs B, C, A, E) (Figure 3). The cluster of cap genes plays a key role in γ-PGA production; however, equivalent importance of all the four *pgs* genes remains questionable. The investigations have concluded more importance of the *pgsC* and *pgsB* genes whereas the *pgsE* gene has been reported as non-essential (Wu et al., 2008; Luo et al., 2016).

Polymerization is an ATP-dependent process where substrate-dependent hydrolysis of ATP leads to the transfer of phosphoryl group to a terminal carboxyl group of elongated γ-PGA. Then, glutamic acid’s amino group conducts a nucleophilic attack on the phosphorylated carboxyl group to form an amide linkage and continues to γ-PGA polymerization at the synthase complex (PgsBCA) active site. *PgsC* and *PgsB* collectively form most parts of complex’s catalytic site whereas *PgsA* contributes to the elongated chain removal from the active site that allows the addition of next monomer and might also participate in γ-PGA transportation. *PgsBCA* activity is known to depend on Mg^2+^. Shorter phospholipid-containing less compact cell membranes could facilitate extracellular γ-PGA transportation (Ashiuchi et al., 2001; Urushibata et al., 2002; Ashiuchi et al., 2004; Candela and Fouet, 2006; Buescher and Margaritis, 2007; Wang et al., 2017).

### 3.3 Depolymerases-based γ-PGA degradation

Troy (1973) was the first to report a depolymerase enzyme that could break down PGA to glutamic acid monomer in the late stationary phase of *Bacillus licheniformis*. This discovery urged several researchers to further investigate this hydrolase enzyme (Kunioka and Goto, 1994; Gross, 1998; King et al., 2000). γ-PGA-synthesizing *Bacillus* strains contain two γ-PGA breaking enzymes (exo- and endo-glutamyl peptidase). Increased depolymerization time results in reduced dispersity as endo-glutamyl peptidase, secreted by *B. licheniformis* and *B. subtilis* in the medium, breaking high molecular weight γ-PGA into fragments (1,000 Da to 20 kDa) (Tsao, 2004). *B. subtilis* is known to contain endo-glutamyl peptidase encoding genes, which have the same orientation as the pgsBCA operon. Their protein contains a cleavage site (30A-E-A32) proximal to the N- and a hydrophobic cluster (10F-L-L-V-A-V-I-I-*CF*-L-V-P-I-M24) (Sebastián et al., 2013).

Some *Bacillus* species secrete peptidase enzymes (GGT (gamma-glutamyltranspeptidases)) under stress conditions or starvation for γ-PGA hydrolysis and utilize released glutamic acid as a nitrogen and carbon source. Gamma-glutamyl-hydrolase encoding *pgdS* gene is present downstream of the pgsBCA operon, which could degrade γ-PGA into two glutamate residues. The *capD* gene belongs to the family GGT, which performs dual functions such as γ-PGA anchorage and depolymerization via cleavage and relocates it to water or an acceptor molecule leading to hydrolysis or transpeptidation. Several studies have reported γ-PGA depolymerase presence and activity in *B. subtilis* NX-2, which carries out γ-PGA depolymerization in the batch culture (Ashiuchi et al., 2003b; Kimura et al., 2004; Candela and Fouet, 2006; Wu et al., 2008; Yao et al., 2009; Morelli et al., 2014; Ogunleye et al., 2015). The extracellular activity of this endo-hydrolase has been reported in the culture, which is encoded by the gene *ywtD* (*pgsS*). During a study, the *ywtD* gene was cloned and expressed in *E. coli* to obtain purified YwtD protein. The enzyme remained active within a pH range of 5–8 and a temperature range of 30–40°C. A reduced γ-PGA molecular mass (1,000 to 20 kDa) was noted at optimal temperature (30°C) and pH (5) and dispersity was alleviated in response to depolymerization time. Extracellular enzyme activity was also observed during the late stationary phase. The process presented a mild technique for controlled molecular mass reduction, which could be employed as a better alternative to chemical and physical degradation methods (Ogunleye et al., 2015).

### 3.4 γ-PGA regulation genes

The ComP–ComA signal transduction system is known to regulate γ-PGA yield in *B. subtilis* (natto) (Tran et al., 2000). Stanley and Lazazzera (2005) have reported an additional two-part system (DegS–DegU, DegQ, and SwrA) as unusual γ-PGA synthesis regulators. The transcriptional effects of ComPA, DegSU, and DegQ occur in response to phase variation signals, quorum sensing, and osmolarity, whereas SwrA activity is considered post-transcriptional. Multiple studies have investigated the DegU and SwrA relationship and revealed that phosphorylated DegU (DegU-P) and SwrA are necessary to completely activate the pgs operon leading to γ-PGA production. The effect of genes on γ-PGA production and pgs transcription remains negligible (Stanley and Lazazzera, 2005; Osera et al., 2009). Contrarily, Ohsawa et al. (2009) demonstrated direct activation of *pgs* expression at a high DegU-P level rather than SwrA and high degQ levels. However, SwrA was still crucial for γ-PGA synthesis under certain conditions. Do et al. (2011) knocked out the *degQ* gene in *B. subtilis* (natto) to identify its role in γ-PGA production. They further isolated suppressor mutants, which could produce γ-PGA in the absence of the *degQ* gene. The results were compared with a domestic *B. subtilis* 168 strain, which cannot produce γ-PGA due to lower biosynthetic *pgs* transcription though is genetically more competent than the wild *B. subtilis* (natto) strain. *degQ* alteration could significantly restrict the γ-PGA production in *B. subtilis* (natto) and downregulate the synthesis of degradation enzymes (Stanley and Lazazzera, 2005; Osera et al., 2009; Do et al., 2011). Thus, the *degQ* gene of *B. subtilis* (natto) is necessary for γ-PGA production (Do et al., 2011).

## 4 Limitations and commercialization strategies of γ-PGA production

The biodegradability, non-toxicity, eco-friendliness, and unique properties have increased the acceptability of microbial biopolymers as compared to synthetic non-degradable materials. Biopolymers are in high demand globally but higher production costs and lower yield limit their commercial usage (Kreyenschulte et al., 2014). Recently, more interest in the commercialization of γ-PGA has been gaining immense attention due to its unique properties and its use in diverse applications. The cost of bioproducts is a crucial aspect in establishing the economic viability of a process, especially when considering its wide range of applications. γ-PGA is an expensive biopolymer and its few milligrams can cost several sterling pounds/or the sentence remain as: few milligrams cost several dollars ($173/100 mg high purity sodium salt γ-PGA; Sigma Aldrich) (Ogunleye et al., 2015). This problem can only be solved by lowering the γ-PGA production cost (Ju et al., 2014). The fundamental research target for industrial application is to optimize the fermentation medium in order to decrease the costs of biopolymer manufacturing. Nevertheless, the production costs of γ-PGA are considerably decreased as they do not need the inclusion of external glutamate (Cai et al., 2023). However, the major economic problem in γ-PGA production is the high cost associated with the media components and the kind of microorganisms used have a significant impact on production costs. The production cost of γ-PGA using L-glutamic acid-independent strains are lower as compared to L-glutamic acid-dependent strains due to the external supply of L-glutamic acid in the fermentation medium. Therefore, glutamate-independent bacteria and genetically modified strains are the most important strategies for commercial production with renewable substrates, which improves the process’s overall economics. Another way to reduce the production cost is the enforcement of a mixed culture strategy. Xu et al. (2005) found that fermentation using mixed cultures provides many benefits over pure cultures. These include a larger yield and the efficient and better use of varied wastes. The fermentation duration and output cost were reduced by co-cultivating *B. subtilis* with *C. glutamicum* utilizing a mixed carbon source of glucose and sucrose. In addition, isolatation and screening osmophilic strains capable of producing γ-PGA at higher sugar concentration results in increased output and productivity in batch fermentation (Luo et al., 2016). Extremophilic microorganisms like halophiles and hyperthermophilic can be investigated for their γ-PGA production. Their ability to grow at higher salt concentrations will also reduce the risk of contamination in non-sterilized fermentation media (Wei et al., 2010; Zeng et al., 2018).

To date, studies have mainly focused on enantiomeric composition, optimization of growth conditions for a higher yield, and achieving the desired γ-PGA molecular mass at a lower cost. The medium of γ-PGA-producing bacteria is of key importance as it can directly affect properties and the production cost of γ-PGA. Another strategy to reduce the production cost of γ-PGA is the utilization of agricultural and food industrial wastes as fermentation substrates. This not only increases productivity but also corresponds with environmentally beneficial methods, making it a green and cost-effective optimization strategy. The usage of lignocellulosic biomass such as rice straw and corncobs can be an appealing carbon source alternative (Huang B. et al., 2011; Zhang et al., 2019b). The carbon source can be further substituted using macroalgae, goose feathers, paper waste, swine, dairy, and chicken manure (Scheel et al., 2019). However, the main restriction with these wastes is the requirment for pretreatment in order to effectively utilize sugars as well as supplying the production media with extra nutrients such as L-glutamic acid, citric acid, peptone, and trace elements, which increases the cost. As a result, for cost-effective synthesis of γ-PGA, it is necessary to have in-depth understanding of diverse waste, their nutritional composition, and their availability for cost-effective synthesis of γ-PGA. This review focuses on recent advances in the biosynthesis of γ-PGA employing seveal *Bacillus* strains for cost-effective production of γ-PGA (Huang J. et al., 2011; Ju et al., 2014; Zhang et al., 2019a). Tang et al. (2015) reported that using rice straw powder as a substrate achieved an increased yield while reducing carbon source consumption. Yong et al. (2011) found that using cow manure as a substrate for γ-PGA production resulted in a yield of 0.0437 g/L of product per gram of substrate at 37°C for 48 h. Also, Li et al. (2020) reported that wastewater from yeast molasses fermentation was repurposed to produce fulvic acid (FA) for PGA production. This method not only substitutes low-cost substrates with inexpensive FA but also reduces pollutants associated with yeast molasses fermentation. Historically, sucrose was the most common substrate, but now non-food raw materials are increasingly utilized, lowering costs and benefiting the environment. Because of its dual impact, it is both cost-effective and an ecologically friendly optimization strategy. While these methods have enhanced γ-PGA output to some extent, they may not entirely solve the strain-imposed restrictions. To ensure stable, high-yield strains, it is necessary to genetically modify the strains to engineer bacteria suitable for industrial production.

## 5 γ-PGA production affecting factors

Microbial γ-PGA production is well developed, but low yield hinders its industrial usage (Luo et al., 2016). Bacterial strains and culturing medium mainly determine the cost of γ-PGA synthesis (Singh et al., 2016). Several *Bacillus* species can produce γ-PGA; however, some strains of *Bacillus licheniformis* and *Bacillus subtilis* have gained more attention for further development.

The development of γ-PGA mass production systems could be a main step toward a feasible solution. Nutrient requirements of various bacterial strains have been investigated for better γ-PGA production. γ-PGA-producing bacteria have been categorized into two groups according to their nutrient requirements such as glutamic acid-independent and glutamic acid-dependent bacteria. Moreover, culture conditions (culture medium, medium pH, nitrogen and carbon sources, aeration, ionic strength, and agitation) also impact PGA quality and productivity (Kunioka and Goto, 1994). A detailed understanding of γ-PGA synthesis-associated genes and enzymes could facilitate bacterial manipulation for higher γ-PGA yield. However, γ-PGA yield has not yet reached the productivity of traditional producers (Gao W. et al., 2016).

### 5.1 Effect of media components on γ-PGA production

Media components and culture conditions are important factors for microbial γ-PGA production as they can impact its characteristics and yield. Nutritional requirements of γ-PGA production vary with L-glutamic acid-independent and -dependent *Bacillus* strains (Kunioka and Goto, 1994; Gross, 1998). Leonard et al. (1958) formulated the Medium E that has been extensively used to culture bacteria (Gross, 1998). Medium E contains high carbon content and mainly comprises L-glutamic acid (20 g/L), glycerol (80 g/L), citric acid (12 g/L), NH_4_Cl, 7 0.0; K_2_HP0_4_ (0.5 g/L), MgS0_4_.7H_2_0 (0.5 g/L), CaCI_2_.2H_2_0 (0.15 g/L), FeCI_3_.6H_2_0 (0.04 g/L), and MnS0_4_.H_2_0 (0.104 g/L). Different media have been explored for enhanced γ-PGA production. L-glutamic acid-producing strains can even produce γ-PGA in L-glutamic acid-lacking media. Different types of fermentation media (wastes and synthetic) have been tested for γ-PGA production (Ju et al., 2014). Optimized medium composition efficiently promotes cell growth and facilitates high accumulation of precursors for γ-PGA production (Gao C. et al., 2016). Engineered strains that can metabolize cheaper substrates offer a cost-effective option for γ-PGA synthesis. Carbon substrates pose direct or indirect impacts on synthetic enzyme activity and γ-PGA production. Glutamic acid, sucrose, glucose, glycerol, and citric acid are common carbon sources with varying metabolic pathways, which influence *Bacillus* species-based γ-PGA production (Moraes et al., 2012; Tork et al., 2015). Carbon source selection mainly depends on bacterial strain for better PGA production.

L-glutamic acid performs a diverse role in PGA-producing strains. Strain and medium components determine the required L-glutamic acid amount for PGA production. Significant interactions of L-glutamic acid with medium components require its optimization, which can directly impact PGA production cost. The L-glutamic acid to PGA conversion rate determines its effective concentration in the medium. Most studies have recommended an L-glutamic acid concentration of 20–30 g/L for PGA production (Cromwick et al., 1996; Du et al., 2005; Kongklom et al., 2015).

PGA production is supported by glycerol and glucose in most glutamic acid-independent strains. Ko and Gross (1998) reported that *B. licheniformis* ATCC 9945A converts glucose to acetyl-CoA and TCA cycle intermediates which form L-glutamic acid to synthesize PGA. Troy (1973) stated glycerol-based stimulation of polyglutamyl synthetase, which catalyzes glutamic acid polymerization to PGA. Wu et al. (2010a) revealed that glycerol not only stimulates PGA production but also reduces its molecular weight in *B. subtilis* NX-2 culture.

Glycerol improves cell permeability more than glucose to facilitate the production and release of γ-PGA (Shih et al., 2001; Du et al., 2005). Moreover, the presence of glycerol in the fermentation medium decreases γ-PGA chain length leading to reduced broth viscosity and enhanced substrate uptake for better cell growth and γ-PGA synthesis. Glycerol can efficiently regulate the molecular weight of γ-PGA (Wu et al., 2010).

Citric acid serves as an effective precursor for PGA production (Jain et al., 2005; Du et al., 2005). Kongklom et al. (2015) have reported the promoter activity of glutamic acid and citric acid for the production of γ-PGA. Goto and Kunioka (1992) revealed that *B. subtilis* IFO 3335-based production strongly depends on the citric acid presence in the fermentation medium. During a study, maltose, xylose, and fructose positively affected cell growth but remained unable to impact γ-PGA production. Zhang H. et al. (2012) noted a maximum γ-PGA yield of 28.3 g/L in the presence of citric acid as compared to other organic acids (oxalic acid, succinic acid, and malic acid).

The addition of metabolic precursors as carbon sources for γ-PGA production might achieve higher yields through enhanced enzyme activity (Nair et al., 2023). Bajaj and Singhal (2009) used *B. licheniformis* NCIM 2324 to screen different TCA cycle intermediates (pyruvic acid, a-ketoglutaric acid, succinic acid, or malic acid) for PGA production and noted a rise in yield (26.12 to 34.98 g/L) after the addition of α-ketoglutaric acid (10 mM) to the medium. Da Silva et al. (2014) reported that α-ketoglutaric acid and L-glutamine added culture medium could enhance the γ-PGA yield of *B. subtilis* BL53 by 20%. Kunioka (1995) demonstrated a higher PGA yield without using other byproducts in an L-glutamine/citric acid medium. Bajaj and Singhal (2009) investigated the impacts of glutamic acid family amino acids (L-proline, L-glutamine, L-ornithine, and L-arginine) and amino acids associated with the PGA biosynthetic pathway (L-aspartic acid and L-alanine) on PGA production and achieved maximum production with 0.5 mM L-glutamine. PGA yield and molecular weight (570 kDa) were further enhanced with the addition of α-ketoglutaric acid and glutamine combination to the medium (Lim et al., 2012).

Biomass materials or by-products can also be converted into high-value γ-PGA. Agro-industrial wastes (rapeseed meal, cane molasses, soybean residue, corncobs, crude glycerol and its hydrolysate, rice straw, and monosodium glutamate) have been investigated for γ-PGA production (Zhang D. et al., 2012; Tork et al., 2015; Zhang et al., 2019). Moreover, carbonaceous substances could replace common carbon sources including dairy products, algae, animal feathers, and chicken manure (Chen et al., 2005; Scheel et al., 2019). Zhang et al. (2019) noted a high γ-PGA yield (65 g/L) in an optimized culture medium supplemented with chicken manure, *glutamic* acid extracts, and soybean residue. Nitrogenous compounds can also impact the γ-PGA production. NH_4_^+^ is known to form γ-PGA monomers with α-ketoglutarate via transamination reaction. The presence of free amino groups is necessary during the γ-PGA synthetic pathway. Glutamate serves as a common nitrogen source for most *B. subtilis* species. Other nitrogenous substances can also replace traditional nitrogen sources. Birrer et al. (1994) optimized the culture medium with fishmeal waste as the nitrogen source and achieved a rise in γ-PGA production up to 25 g/L.

Inorganic salts (MnSO_4_ and CaCl_2_) can significantly affect the stereochemical composition and yield of PGA. CaCl_2_ addition to the medium can reduce culture broth’s viscosity and increase extracellular glutamic acid consumption (11.4%) resulting in a higher yield of PGA than controls (Shih and Van, 2001; Huang J. et al., 2011). CaCl_2_-based enhanced enzymatic activities of ODHC (2-oxoglutarate dehydrogenase complex), GDH (glutamate dehydrogenase), and ICDH (isocitrate dehydrogenase) have been reported in the PGA biosynthesis pathway, which directed 2-oxoglutarate for higher glutamic acid production (Bajaj and Singhal, 2009; Huang J. et al., 2011). Metal ion cofactors help in carbon source utilization. Mn_2_^+^ could considerably improve glutamate racemase activity leading to higher yield (9.25 to 28.42 g/L) of γ-PGA (Wu et al., 2006; Kongklom et al., 2015). Sodium salts in the culture medium can regulate γ-PGA yield and molecular weight in extremophilic bacteria and halotolerant strains (Wei et al., 2010). In addition to phosphorylation-based derivatives of γ-PGA, sulfonation or esterification can be directly achieved by adding phosphoric acid to the growth media. γ-PGA derivatives are more potent than native γ-PGA with diverse applications (Ashiuchi, 2010). Thus, exogenous culture additives are effective in γ-PGA production.

### 5.2 Impact of fermentation conditions on γ-PGA production

Fermentation conditions can be modified for higher γ-PGA productivity, concentration, and yield (Table 1). Suitable culture conditions (temperature, pH, inoculation amount, and oxygen content) can efficiently enhance γ-PGA yield (Bajaj and Singhal, 2009). pH is an important γ-PGA biosynthesis factor as glutamate-involving reactions exhibit pH sensitivity (Luo et al., 2016). pH control promotes glutamate utilization for better γ-PGA yield. Cromwick et al. (1996) stated the dramatic impact of pH on PGA production and found that pH reduction (7.4 to 6.5) caused a four-fold rise in PGA yield with *B. Iicheniformis* (Bajaj and Singhal, 2009). *Bacillus* species are mostly aerobic and mesophilic, which flourish in the optimum temperature range and affect the synthesis of γ-PGA (Zeng et al., 2014). However, thermoacidophiles can survive at high temperatures by maintaining thermal stability. Cromwick et al. (1996) investigated the role of agitation and aeration in carbon usage, PGA production, and biomass production. Higher oxygen supply to *B. licheniformis* ATCC 9945A could double its dry cell weights. Citric acid and L-glutamic acid depleted more quickly at high aeration and increased PGA yield (23 g/L) compared to the yield (6.3 g/L) at a low oxygen supply. A high aeration rate led to a higher rate of molecular weight reduction with time in comparison to a low aeration rate. This can be associated with depletion of the carbon source and higher activity of PGA depolymerases at a high aeration rate (Birrer et al., 1994).

**Table 1:** Strains, key nutrients of fermentation media, methods, conditions, and yields of γ-PGA.

Isolate	Nutrients of fermentation media	Methods and key conditions	Yield (g/L)	References
*B. subtilis* ZJU-7	Glucose, l-glutamate, yeast extract, NaCl, CaCl_2_, MgSO_4_, MnSO_4_	Bioreactor, 300–800 rpm, pH 6.5, 37°C	101.1	Huang et al. (2011)
*B. subtilis* NX-2	Glutamate, (NH_4_)2SO_4_, K_2_HPO_4_, MgSO_4_, MnSO_4_, and hydrolysis of rice straw	Bioreactor, 400 rpm, initial pH 7.0, 32°C	73.0	Tang et al. (2015)
*B. subtilis* MJ80	Glutamic acid, starch, urea, citric acid, glycerol, NaCl, K2HPO_4_, MgSO_4_, MnSO_4_	Bioreactor, 37°C, 150 rpm, initial pH 7.0	68.7	Ju et al. (2014)
*B. subtilis* CCTCC202048	Swine manure, soybean cake, wheat bran, glutamic acid, citric acid	Flasks, shaking at 180 rpm, and initial pH 9.0, 37°C for 48 h	60.00	Chen et al. (2005)
*B. subtilis* NX-2	Cane molasses and monosodium glutamate waste liquor	Bioreactor, 400 rpm, 32°C, pH 7.0	52.1	Zhang D. et al. (2012)
*B. subtilis* RKY3	Glycerol, glutamic acid yeast extract, K_2_HPO_4_	Flasks, shaking at 200 rpm, pH 6.5, and 38°C for 48 h	48.5	Jeong et al. (2010)
*B. subtilis* (natto) MR-141	Maltose, soy sauce, sodium glutamate	Flasks, shaking at 400 rpm and pH 8 for 72 h at 40°C	35.00	Ogawa et al. (1997)
*B. subtilis* NX-2	Glutamic acid, glucose yeast extract	Flasks, shaking at 220 rpm, pH 7.0, and 37°C for 24 h	30.20	Xu et al. (2005)
*B. subtilis* 242	Cane molasses, l-glutamic, corn steep liquor	Flasks, shaking at 220 rpm, pH 7.0, and 37°C for 48 h	32.14	Li S. et al. (2022)
*B. subtillis* HB-1	Glutamate, yeast extract, NaCl, MgSO_4_, xylose, or corncob fibers hydrolysate	Bioreactor, 500 rpm, 37°C, initial pH 6.5, fed-batch	28.15	Zhu et al. (2014)
*B. subtilis* GXG-5	Glucose, ammonium nitrate	Flasks, shaking at 200 rpm, pH 6.5, 37°C for 34 h, 50°C	19.5	Zeng et al. (2017)
*B. sonorensis* 44	Glycerol, yeast extract, α-ketoglutaric acid	Flasks, shaking at 200 rpm, pH 6.5, 37°C for 72 h, 30°C	11.84	Azarhava et al. (2020)
*B. licheniformis* NCIM 2324	Soybean meal, citric acid, glutamic acid (NH_4_)_2_SO_4_, glycerol, L-glutamine, c-ketoglutaric acid	Flasks, shaking at 200 rpm, and pH 7 ± 0.2 for 96 h at 37 ± 2°C	98.64	Bajaj et al. (2008)
*B. licheniformis* CGMCC NO. 23967	Monosodium glutamate, sugarcane molasses, yeast extract	Flasks, shaking at 200 rpm, pH 6.5, for 72 h, 37°C	76.85	Guo et al. (2023)
*B. licheniformis* P-104	Glucose, sodium glutamate, sodium citrate, (NH_4_)_2_SO_4_, MnSO_4_, MgSO_4_, K_2_HPO_4_	Bioreactor, 500 rpm, 37°C, pH 7.0, fed batch	41.6	Zhao et al. (2013)
*B. licheniformis* NCIM 2324	Glycerol, l-glutamic acid, citric acid, (NH4)2SO4, K2HPO_4_, MgSO_4_, MnSO_4_	Flask, shaking at 200 rpm, 37°C, initial pH 6.5	35.75	Bajaj and Singhal (2009)
*B. methylotrophicus* SK19.001	Glucose, yeast extracts, MgSO_4_, K_2_HPO_4_, MnSO_4_	Flask, shaking at 200 rpm, initial pH 7.2, 37°C	35.34	Peng et al. (2015)
*B. licheniformis* TISTR 1010	Glucose, citric acid, NH4Cl, K_2_HPO_4_, MgSO_4_, CaCl_2_, MnSO_4_, NaCl, Tween 80	Fermenter, 300 rpm, initial pH 7.4, 37°C	27.5	Kongklom et al. (2015)
*B. licheniformis* ATCC 9945	Glutamic acid, glycerol, acid, NH_4_Cl	Flasks, shaking at 250 rpm at pH 6.5 for 96 h	23.00	Cromwick et al. (1996)
*B. licheniformis* A35	Glucose, MnSO_4_, ammonium chloride	Flask, slow shaking, initial pH 7.2 at 30°C for 24 h	8.1	Cheng et al. (1989)

To obtain high γ-PGA production, the oxygen supply can be maintained by placing culture in the fermenter or adding an oxygen-carrying agent (Da Silva et al., 2014). The inoculation amount depends on culture conditions, culture composition, and bacteria (Meissner et al., 2015). An inappropriate inoculum concentration results in nutrient competition or cell growth inhibition, which ultimately leads to reduced γ-PGA production. Studies have focused on the integration and optimization of culture conditions to reduce production cost and fermentation time for sustainable γ-PGA yield and productivity. Several techniques such as continuous culture, fed-batch, batch, and symbiotic fermentation (co-cultured strains or mixed strains) have been studied for better γ-PGA productivity (Ashiuchi, 2013; Hirasawa and Shimizu, 2016; Jose et al., 2018). The yield of γ-PGA was increased to 2.19 g/L in a fermenter using a fed-batch glucose supply (Huang B. et al., 2011).

### 5.3 Improved cell membrane permeability for enhanced γ-PGA secretion

Intracellular glutamic acid combined with a membranous mechanism synthesizes extracellular PGA. Therefore, intracellular substrate amount is a crucial factor for γ-PGA biosynthesis, and its higher level could more efficiently produce this polymer (Wu et al., 2008). Cellular stimulation for γ-PGA secretion also alleviates the stress linked to the accumulation of intracellular γ-PGA and substrate leading to enhanced γ-PGA formation. Improved cell membrane permeability increases extracellular substrate consumption and PGA secretion. The addition of glycerol, Dimethyl sulfoxide (DMSO), or Tween 80 addition to the medium enhances extracellular substrates’ uptake and γ-PGA secretion (Du et al., 2005; Wu et al., 2008). During a study, DMSO and Tween 80 supplemented medium improved the extracellular glutamate uptake (3.88 mmol/g DCW/h and 3.06 to 3.92). Wu et al. (2010) have revealed that DMSO and Tween 80 could increase carbon sources’ utilization and affect cell membrane permeability to regulate substrate availability.

### 5.4 Genetic manipulation

Most native γ-PGA producers are associated with *Bacillus* species; particularly, *B. licheniformis* and *B. subtilis* are widely applied in industrial applications (Bajaj et al., 2009). Fermented soybean products and certain environments are the main sources of γ-PGA-producing strains. However, the lower rate of substrate conversion and higher production cost of glutamate-dependent strains emphasize the utility of glutamate-independent strains. Genetic manipulation is an established method for the regulation of γ-PGA synthesis (Cao et al., 2018). Genetic manipulation facilitates the development of genetically engineered strains containing construction plasmid and knockout genes. Genetic manipulation in *Bacillus* species can indirectly or directly inhibit or promote enzyme expressions to influence γ-PGA biosynthesis and breakdown.

Genetic manipulation in the γ-PGA synthesis pathway of native strains is a common technique. Zhang et al. (2015) reported that the individual deletion of *gudB* (glutamate dehydrogenase) or *rocR* (transcriptional regulating) genes in *B. amyloliquefaciens* LL3 efficiently increased γ-PGA yield. The deletion of *gudB, fadR, aspB, lysC, pckA, rocG*, and *proAB* genes in *B. amyloliquefaciens* strain NK-A6 partially blocks the downstream metabolic pathways. *B. amyloliquefaciens* NK-A7 can be genetically manipulated by inserting a strong PC2up promoter to enhance the NADPH level. *B. amyloliquefaciens* NK-A11 is genetically manipulated by deleting itu and srf operons. These engineered bacteria have been reported to, respectively, produce 4.84 g/L, 7.53 g/L, and 6.46 g/L γ-PGA, which are higher than original *B. amyloliquefaciens* LL3 strains (Gao et al., 2019). A gene cluster (*ywsC-ywtAB* or *PgsBCA*) integrated with a regulating *SwrA* gene enhances intracellular SwrA and DegU-P levels and γ-PGA production. Similarly, chromosomes embedded with *PgsBCA* via a strong promoter can significantly improve γ-PGA yield. The unavailability of external nutrients could lead to γ-PGA hydrolysis into nitrogen or carbon sources by its producing strains (Cao et al., 2018). γ-PGA hydrolase repression through genetic engineering could mitigate the γ-PGA production loss and alter its molecular weight (Scoffone et al., 2013; Mitsunaga et al., 2016). γ-PGA hydrolase expression has been manipulated in various studies by adopting the gene knockout technique. Kimura et al. (2004) have reported higher γ-PGA molecular weight and comparable yield of the *ggt*-deleted mutant *B. subtilis* NAFM5 with wild strains. The deletion of the *cwlO* gene in *B. amyloliquefaciens* and *B. subtilis* is known to promote γ-PGA yield with higher molecular weight compared to the wildtype strain, whereas deletion of *pgdS* gene only caused a minor increase in molecular weight (Mitsui et al., 2011; Zhao et al., 2013). However, a combined deletion of the *ggt*, *pgdS*, and *cwlO* genes in *B. amyloliquefaciens* and *B. subtilis* strains significantly increased γ-PGA yield (93%) in comparison to wildtype strains (Ohsawa et al., 2009; Tian et al., 2014). Feng et al. (2015) reported a minor improvement in the *B. amyloliquefaciens* NK-E10 strain’s γ-PGA production capability after the deletion of the *luxS* gene. Furthermore, improved carbon flux conversion to γ-PGA production, elimination of undesired precursor or by-products, and inhibition of drain energy pathways can enhance γ-PGA purity and production (Nair et al., 2023). Extra ATP can positively affect carbon metabolism for improved γ-PGA production (Peng et al., 2016). Polysaccharides are major γ-PGA contaminants and by-products, which utilize a considerable amount of metabolic energy and carbon sources (Cao et al., 2018). Genetic mutation can knock out lipopolysaccharide and exopolysaccharide synthesizing genes to achieve pure γ-PGA (Feng et al., 2015; Tian et al., 2019). The removal of lipopolysaccharides from the *B. amyloliquefaciens* NK-E5 culturing medium increased γ-PGA purity to 95% compared to lipopolysaccharides containing medium (Feng et al., 2015). Acetate and lactate negatively impact the bacterial cell growth. Thus, mutants with knocked-out acetate and lactate synthesis genes yielded pure γ-PGA. However, the elimination of glutamate-consuming pathways remained unable to promote γ-PGA yield because the gene manipulation led to a metabolic imbalance with related cellular pathways (Feng et al., 2015; Cao et al., 2018). The synthesis of energy-consuming substances (antibiotics) coincides with γ-PGA production. The repression of such substances via gene knockout could improve γ-PGA production compared to native strains (Gao et al., 2019).

Heterologous or homologous hosts’ recombinant expression is also an efficient technique for enhancing γ-PGA production. *Corynebacterium glutamicum* and *Escherichia coli-*based genetic manipulation is referred to as heterologous expression whereas homologous expression is recombined by *Bacillus*. The introduction of xylose-induced plasmid pWH1520 with pgsBCA operon into *B. subtilis* MA41 disrupts the native *pgsBCA* gene and facilitates the successful expression of γ-PGA synthetase (Ashiuchi et al., 2006). Energy-saving NADPH-GDH pathway-containing plasmid in *B. amyloliquefaciens* NK-1 could elevate the γ-PGA production by 9%. γ-PGA yield is mainly influenced by genetic expression patterns such as inducible and constitutive expressions (Scoffone et al., 2013; Chen et al., 2018). Inducible expression is capable of rapidly accumulating γ-PGA. During a study, simultaneous cloning of racE of *B. amyloliquefaciens* LL3 and pgsBCA operon of *B. licheniformis* NK-03 was carried out into an induced plasmid followed by co-expression in *E. coli* JM109 for the microbial production of γ-PGA. Jiang et al. (2006) recombined *Geobacillus toebii* gene’s (D-AAT (D-amino acid aminotransferase)) constitutive promoter (PHCE) and expressed it in *E. coli*/ pCOpgs for yielding γ-PGA (3.7 g/L) in an optimized medium. Native L-glutamate-yielding *C. glutamicum* could also serve as a suitable host for γ-PGA’s recombinant production. Cao et al. (2018) revealed that heterologous expression of PgsBCA operon in *C. glutamicum* yielded 0.7 g/L of γ-PGA even in a glutamate-deficient medium.

### 5.5 Extraction, recovery, and purification of γ-PGA

One of the major hurdles for scaling up high purity γ-PGA manufacturing is the downstream process. Several downstream procedures are critical for recovering and characterizing γ-PGA produced in the fermentation medium (Nair et al., 2023). Extracellular γ-PGA synthesis in *Bacillus* species facilitates its recovery and purification. The molecular weight and yield of γ-PGA are key factors, which influence its purification and recovery cost (Markus et al., 1985; Wu et al., 2010). γ-PGA recovery goals mainly include: (i) concentrating the fermentation broth/extract to obtain a stable solid form of microbial product, which is easy to store, handle, transport, and re-dissolve/dilute for specific applications; (ii) purification to mitigate non-polymer solids (salts or cells) and improve its functional performance, taste, color, and odor; (iii) deactivation of contaminating enzymes; and (iv) alteration of chemical properties to amend functional performance, handling properties of solid dried product, dispersion, and solubility rate (Smith and Pace, 1982). PGA is recovered by centrifuging (20,000×*g* for 15 min) the fermentation broth, which removes cells and simplifies the purification process. The dilution of highly viscose γ-PGA and pH adjustment to 3 helps in reducing viscosity and removing producing strains (Do et al., 2001).

#### 5.5.1 Precipitation by solvents

By changining the materials into an insoluble form, the precipitation process converts the desired products (chemical compounds) from a solution into a solid form or crystals. To perciptate γ-PGA from fermented broth, alcohols such as methanol or ethanol are usually used. Methanol or ethanol (4: 1) is used to precipitate, concentrate, and partially purify γ-PGA. Then, crude PGA is re-dissolved in distilled water (100 to 200 w/v) and centrifuged (5,000 g for 15 min) to discard the remaining solids. Finally, the diluted PGA solution is dialyzed or ultra-filtered to remove low-molecular-weight impurities and salts followed by freeze drying to a stable solid form. However, PGA usage in drug delivery requires further purification to isolate fractions of specific molecular weight (Goto and Kunioka, 1992; Tork et al., 2015). Dissolved γ-PGA significantly increases the viscosity of fermentation broth, which complicates cell separation from the broth. However, broth dilution and pH modification to 3 could facilitate the separation process (Do et al., 2001). A lower pH helps in alleviating cells’ zeta potential and viscosity and facilitates flocculation (Do et al., 2001). Organic solvent-based γ-PGA precipitation is the commonly used method where ethanol is the most preferred precipitator from a cell-free broth (Do et al., 2001; Nair et al., 2023). Three to four times the broth volume, cold 2-propanol, methanol, and ether and n-propanol mixture (1:1 v/v) can also be used for γ-PGA precipitation (Kubota et al., 1993; Birrer et al., 1994; Kreyenschulte et al., 2014). γ-PGA containing cell-free broth can be concentrated by membrane-based ultrafiltration with a 500 kDa molecular weight cut-off, which reduces the amount of organic solvents (Do et al., 2001). However, non-specific solvent precipitation could lead to coprecipitation of other proteins as well, which might require further purification. Moreover, this type of precipitation is not quantitative and almost 15% of the product could be lost in the solution (Manocha and Margaritis, 2010; Kreyenschulte et al., 2014). Diafiltration can directly recover PGA from the broth. The use of an ultrafiltration membrane only allows the permeation of dissolved polymers whereas the cells are screened out. The continuous addition of water to lower the viscosity results in an increased volume of permeation and product dilution. Generally, viscous solutions slow the membrane-based filtration. Therefore, a novel method has been devised for a continuous membrane-based cell recycling system-integrated fermentation process for γ-PGA recovery. Poly vinylidene fluoride (PVDF) hydrophilic microfiltration membranes (0.45 μm) have been used in crossflow modules for cell recycling and separation (Kumar and Pal, 2015). Polyethersulfone (PES-5) membranes have also been used to concentrate cell-free broth by ultrafiltration. However, due to a simple methodology without any specific equipment, cold ethanol is the preferred γ-PGA precipitation technique where ethanol can be completely removed from the final product without potential residues. Pretreatment of cell-free broth with proteases and acid hydrolyzes carbohydrate polymers and contaminant proteins, which can otherwise co-precipitate with target γ-PGA.

#### 5.5.2 Precipitation by metal ions

Recently, a precipitation method using different metal ions such as CuSO_4_, FeCl_3_, AlCl_3_, and MnSO_4_ has been used for γ-PGA precipitation in aqueous solutions as well as from fermented broth. In this method, it was noticed that with the addition of 500 mM CuSO_4_ to the supernatant of the fermentation broth, up to 95% of γ-PGA was precipitated and recovered. To achieve purified γ-PGA, the formed Cu^2+^-γ-PGA complex was collected by centrifugation and re-dissolved in Ethylenediamine tetraacetic acid (EDTA), followed by dialysis against ultra pure water and lyophilization. The recovery percent of γ-PGA using metal ion-induced precipitation is 85% compared to 82% for precipitation by alcohol. Similarly, the co-precipitation of proteins is just 3% with this strategy compared to 50% using alcohol precipitation, indicating its higher selectivity (Kreyenschulte et al., 2014; Luo et al., 2016; Scheel et al., 2019; Nair et al., 2023). However, toxic metal ions can contaminate the environment and final product (McLean et al., 1990; Luo et al., 2016).

#### 5.5.3 Ultrafiltration

Ultrafiltration is another successful recovery method for γ-PGA since it eliminates the need for a solvent in the downstream process. It can maintain the macromolecules in solutions by using hollow fiber membranes to concentrate high molecular weight γ-PGA. In this method, the fermentation culture containing γ-PGA was centrifuged at 10,000 rpm for 30 min, and the resulting cell-free liquid was subsequently concentrated by ultracentrifugation. The loss of γ-PGA was insignificant at the 30 to 100 kDa membrane mol. wt. cut-off (MWCO), although the flow rate was modest. Nevertheless, the flow was comparatively greater with a MWCO of 500 kDa, resulting in a little 3% loss in γ-PGA. The concentration of the cell-free supernatant is greatly affected by the pH in ultrafiltration, as a change in the structure of γ-PGA causes a significant loss at lower pH levels (Gunawan et al., 2011).

## 6 Identification and characterization of γ-PGA

### 6.1 Determination of molecular weight

γ-PGA molecular characterization is necessary to understand its properties and potential applicability (Sung et al., 2005b). Gel permeation chromatography (GPC) is performed to measure the polydispersity and molecular weight of PGA. Polydispersity and average molecular weight alter with the PGA-producing strain and culturing conditions. GPC employs various mobile phases for calibration in reference to different molecular mass standards (Birrer et al., 1994). PGA molecular weight depends on bacterial strains (Shih and Van, 2001), medium components (Birrer et al., 1994; Kunioka and Goto, 1994; Wu et al., 2008), and culturing conditions (Cromwick et al., 1996). The molecular complexity and instability of bacterial cultures complicate the synthesis of highly homogenous PGA. However, *Bacillus* species produce γ-PGA within a molecular mass range of 10^5^ to 10^6^ Da, and it can differ with the application mode (Shih and Van, 2001). The reduction of molecular mass is a key step in γ-PGA synthesis for drug delivery. Alkaline hydrolysis, ultrasonic degradation of medium composition, and enzymatic or microbial degradation techniques are adopted for PGA molecular mass reduction (Shih and Van, 2001). Ultrasonic degradation is the most effective option to reduce the dispersity and molecular mass of biosynthesized PGA without affecting its chemical composition (Graciela et al., 2000). Richard and Margaritis (2006) used the *B. subtilis* IFO 3335 strain for the *in situ* γ-PGA depolymerization in cell-free fermentation broth. GPC and intrinsic viscosity correlations were employed, which revealed a molecular mass reduction from ~46,106 to 5.56104 Da in 144 h. Yao et al. (2009) revealed reduced γ-PGA dispersity as a function of hydrolysis time. Enzymatic degradation is a better technique for achieving the required γ-PGA molecular mass in a controlled way.

### 6.2 Analysis of amino acids

Amino acids containing only glutamic acid represent γ-PGA purity (Shih and Van, 2001). Thin-layer chromatography (TLC) or amino acid analyzers are used for amino acid analysis. Amino acid analysis is carried out by hydrolyzing purified γ-PGA with HCl (6 M) at 100°C for several hours in an airtight tube followed by the evaporation of HCl. Then, water is used to hydrolyze the final product, and amino acid content is analyzed by TLC (Yokoi et al., 1995). TLC is performed on a cellulose plate with different solvent systems (butanol/acetic acid/water (3:1:1, w/w/w) and 96% ethanol/water (63:37, w/w)), and ninhydrin (0.2% in acetone) is sprayed for amino acid identification (Yokoi et al., 1995; Shih et al., 2001). Purified γ-PGA is analyzed by HPLC (high-performance liquid chromatography) according to the methodology of Aboulmagd et al. (2000). Briefly, γ-PGA is hydrolyzed in HCl (6 N, 1 mg/100 mL) in a closed and evacuated tube at 95°C for 12 h, lyophilized, dissolved in distilled water (equal volume), and subjected to HPLC. Pure amino acids are used to chromatographically calibrate γ-PGA (Goto and Kunioka, 1992; Aboulmagd et al., 2000; Shih et al., 2001).

#### 6.2.1 Fourier-transform infrared spectroscopy

Fourier-transform infrared (FTIR) spectroscopy is commonly applied for γ-PGA identification. FTIR spectroscopy generates γ-PGA IR spectra containing peaks related to its specific bonds. Ho et al. (2006) stated that the IR spectra of γ-polyglutamate salts in KBr pellets and γ-PGA (free acid form) indicated a distinct strong amide absorption (~1,620–1,655 cm^−1^), a strong OH (hydroxyl) absorption (~3,400–3,450 cm^−1^), a weaker C=O (carbonyl) absorption (~1,394–1,454 cm^−1^), and strong characteristic C–N group absorption within a range of 1,085 to 1,165 cm^−1^. Absorption peaks between 2,900 and 2,800 cm^−1^ represented characteristic aliphatic N–H stretching whereas peaks at 1,390–1,450 cm^−1^ and 1,600–1,660 cm^−1^ corresponded to the C=O and amide groups, respectively.

#### 6.2.2 Nuclear magnetic resonace (NMR) spectroscopy

The degree of γ-PGA esterification and homogeneity is often determined by ^1^H- and ^13^C-NMR spectroscopy (Birrer et al., 1994; Borbély et al., 1994). Chemical shifts of NMR spectra are measured with reference to known standards.

## 7 Quantification of γ-PGA content in supernatants of crude fermentation

Impurities in γ-PGA hinder its quantification in most of the current methods. Yu et al. (2021) developed a copper ion-based complex for the accurate and rapid quantification of γ-PGA content and common impurities in fermented broth (proteins, glucose, and glutamic acid). The results revealed that only γ-PGA precipitated with copper ions, which linearly correlated with the precipitated amount. The results had an accuracy and precision of 95.82 and 99.29%, which is significantly higher than other methods (weighing and UV). Therefore, copper ion complex formation is a convenient method to assess contents in crude biological samples.

## 8 Applications of γ-PGA

γ-PGA unique properties (non-toxicity, biodegradability, water-solubility, edibility, thickness, moistness, non-immunogenicity, and antimicrobial and antioxidant potential) favor its broad range applications in food, cosmetics, medicine, agriculture, and bioremediation fields (Richard and Margaritis, 2001; Sung et al., 2005a; Candela et al., 2009; Akagi et al., 2010; Ashiuchi, 2010; Bajaj and Singhal, 2011b; Ashiuchi et al., 2013; Ogunleye et al., 2015; Luo et al., 2016; Wang et al., 2022).

### 8.1 Medical and pharmaceutical applications of γ-PGA

γ-PGA has a wide range of applications in pharmaceutical manufacturing including delivery systems, tissue engineering, gene carriers, and therapeutic and immunological effects. It can significantly reduce drug toxicity and improve drug efficiency in combination with other matters (Luo et al., 2016; Zhan et al., 2018; Cai et al., 2023). However, molecular weight is a decisive factor for drug-delivery properties such as the controlled and delayed release of drugs (Luo et al., 2016). Γ-PGA has also been applied as a novel biological adhesive that was formed by its chemical cross-linking with other compounds (Lee et al., 2020). Moreover, γ-PGA serves as a good adjuvant by inducing a higher vaccine antigens’ immunogenicity (Nguyen et al., 2019). Γ-PGA is known to protect probiotics and Caco-2 cells against oxidative damage as well (Csikós et al., 2018).

#### 8.1.1 γ-PGA applications as drug carrier and anticancer agent

The biodegradability and biocompatibility of γ-PGA favor its application as a drug delivery carrier. The side chains of γ-PGA contain carboxyl groups for the conjugation of chemotherapeutic agents, which facilitate the drug solubility and administration. The PGA-drug conjugate reaches the tumor sites and gradually releases the drug with its biodegradation. Glutamic acid, a breakdown product of PGA, can enter the normal cellular metabolism (Singer, 2005). Several conjugants of PGA and anticancer agents have been studied. Li et al. (1998) devised a PGA–paclitaxel conjugate to enhance the antitumour efficacy, stability, and water solubility of paclitaxel. The conjugate exhibited significantly higher antitumour activity against ovarian and breast cancers in human tumor xenografts and murine models as compared to regular paclitaxel treatment. Tumor cell uptake of the PGA–paclitaxel conjugate was noted to be five-fold higher than paclitaxel. The study further elaborated that the PGA–paclitaxel conjugate did not support tubulin polymerization and growth and viability of the Taxol-dependent CHO cell line. Singer (2005) prepared another γ-PGA conjugate with paclitaxel known as paclitaxel poliglumex. It presented more advantages than standard paclitaxel administration such as water solubility, better stability in plasma to mitigate systemic exposure to free paclitaxel, smaller distribution volume, longer elimination and distribution phases, and improved selectivity through higher retention and accumulation in tumor tissue. During a study, oral administration of high molecular mass (26,106 Da) γ-PGA led to the induction of (NK)-cell-mediated antitumour immunity in mice suffering from MHC (major histocompatibility complex) class I-deficient tumours. The antitumour immunity mechanism was found to be associated with γ-PGA-based activation of NK cells rather than direct cytotoxicity. The results revealed comparable antitumour effects of γ-PGA (26,106 Da) against B16 tumours in mice (C57BL/6) with b-glucan (curdlan) that exerts immunomodulating antitumour effects by activating NK cells. These findings advocate γ-PGA application in the immunotherapy of cancers (Kim et al., 2007). Based on an *in vitro* study, Shin et al. (2015) suggest that γ-PGA induces apoptosis in TPA-induced HT-29 human colorectal cancer cells and enhances apoptosis in colorectal cancer cells. Zhang et al. (2018) highlighted that the PGA–Asp–maleimide–cisplatin–peptide complex (PAMCP) reduced the side effects of cis-Dichlorodiamineplatinum (CDDP) and exhibited stronger anti-tumor effects. Therefore, PAMCP presented the potential to be a safe and effective anticancer pharmaceutical formulation for future clinical applications. Tsao et al. (2011) designed a polyelectrolyte complex (PEC) for wound dressing, which contained chitosan (cationic polyelectrolyte) and γ-PGA (anionic polyelectrolyte). These chitosan/γ-PGA PECs provided ample moisture content where γ-PGA more efficiently reduced dehydration risk than regular chitosan. Animal model-based investigations revealed that chitosan/γ-PGA PEC-treated wounds healed more rapidly than untreated wounds. Chitosan/γ-PGA PECs efficiently suppressed inflammatory cells in comparison to regular chitosan, which confirmed the anti-inflammatory potential of γ-PGA. Chitosan/γ-PGA PEC-treated wounds displayed more development of keratin than controls and regular chitosan-treated wounds. After healing, the chitosan/γ-PGA PECs were easily removed from the wound surface and did not damage the regenerated tissues. Thus, chitosan/γ-PGA PECs proved an effective wound dressing material. Ryu et al. (2011) developed γ-PGA and L-phenylalanine nanoparticles (γ-PGA-Phe NPs) and used them for the treatment of retinal diseases in *in vivo* and *in vitro* investigations. γ-PGA-Phe NPs and Texas red-labelled ovalbumin were applied in the eye to study the NPs’ dynamics. Similarly, dexamethasone-containing NPs were administered for the *in vivo* immunosuppressive treatment of microglia and macrophages against various pathological retina disorders. γ-PGA-Phe NPs effectively regulated inflammatory phagocytic cells in the retina under pathological conditions. The results also demonstrated γ-PGA-Phe NPs’ potential as long-term drug delivery carriers in the damaged retina. γ-PGA-Phe NPs proved more advantageous than triamcinolone acetate steroid, which is commonly administered for the treatment of retinal disorders. γ-PGA-Phe NPs can help in avoiding steroid-induced post-capsular cataract formation and glaucoma. Moreover, direct steroid application is toxic to retinal neurons whereas γ-PGA-Phe NPs specifically target microglia and macrophages and thus minimize steroid-associated complications (Ryu et al., 2011).

#### 8.1.2 γ-PGA applications in tissue engineering

Polymer-based hydrogels have numerous applications in medicine (e.g., drug delivery, tissue engineering, wound dressings), cosmetics, and various industrial uses (e.g., contact lenses, hygiene products) (Okay, 2010; Jia et al., 2021; Yi et al., 2021).

Chitosan is the most suitable polymer in tissue engineering among biodegradable polymers. However, the mechanical strength of its scaffolds must be improved for better utility in tissue engineering. Hsieh et al. (2005) inducted PGA into chitosan matrices to enhance the rate of water absorption, surface hydrophilicity, mechanical strength, and composite biomaterial absorption rate. These composite matrices effectively improved cell proliferation and attachment.

#### 8.1.3 γ-PGA applications as antimicrobial agent

The development of novel bactericidal materials requires continuous efforts in medicine, healthcare, and food packaging sectors. The use of hydrogels based on γ-PGA is gaining populariy and several approaches have been proposed such as the bonding of the carboxyl group to nanoparticles and the development of ionic hydrogels based on gelatin and γ-PGA. Tajima and Sukigara investigated the mechanical and bactericidal properties of non-woven γ-PGA cross linked with oxazoline (Tajima and Sukigara, 2012; Wang et al., 2012; Garcia et al., 2014; Gao C. et al., 2016). Currently, hydrogels are widely studied for wound dressings. However, wound healing is often hindered by bacterial infection (Yu et al., 2023). The preparation of γ-PGA-based hydrogels, the bonding of carboxyl group to nanoparticles, and development of γ-PGA and gelatin-based ionic hydrogels are gaining popularity (Garcia et al., 2014; Gao C. et al., 2016; Chen et al., 2023; Yu et al., 2023). γ-PGA-derived biocompatible antimicrobial hydrogels (TCS (Triclosan), CHX (Chlorhexidine), and PHMB (Polyhexamethylene biguanide)) have been prepared using electrospun nanofibers and bulk, which displayed promising antimicrobial efficacy against gram-positive *staphylococcus aureus* and gram-negative *E. coli* (Patel et al., 2007; Vedadghavami et al., 2017; Kasbiyan et al., 2022). The antimicrobial activity of chitosan-γ-PGA polyelectrolyte complex hydrogels and γ-PGA-coated magnetite particles have been reported against *S. aureus* and *E. coli* (Tsao et al., 2010; Inbaraj et al., 2011). However, the antimicrobial potential of pure γ-PGA largely remains unknown. Lee et al. (2013) have reported the antibacterial efficacy of γ-PGA from *B. subtilis* D7 against *Klebsiella pneumoniae, Salmonella typhimurium, Listeria monocytogenes, Escherichia coli,* and *Staphylococcus aureus*. Antibacterial activity was significantly higher against gram-positive bacteria compared to gram-negative bacteria. Different *Bacillus megaterium* UP47-purified γ-PGA concentrations have also demonstrated antimicrobial properties against gram-negative and gram-positive pathogens (*pseudomonas aeruginosa, Klebsiella pneumoniae, shigella dysenteriae, Escherichia coli,* and *staphylococcus aureus*) (Lee et al., 2014; Ajayeoba, 2019; Odeniyi and Omoleye, 2021). Odeniyi and Omoleye (2021) observed that even a low γ-PGA concentration (0.5 mg/mL) could successfully inhibit overnight cultures of *E. coli, S. dysenteriae, P. aeruginosa,* and *S. aureus.* However, *K. pneumoniae* activity was inhibited at a γ-PGA concentration of ≥1.0 mg/mL. Lee et al. (2014) stated that a 1% concentration of γ-PGA-adsorbed discs effectively inhibited microbial growth. A high concentration of γ-PGA (150 mg/mL) has been reported to exert significant inhibitory impacts on *S. aureus* (16.6 mm to 22.5 mm). The nature of microbial pathogens and γ-PGA anions determine its antimicrobial potential. These factors facilitate γ-PGA binding with cell wall components leading to cell disruption (Bajestani et al., 2018; Ajayeoba et al., 2019). The pathogenic bacteria protect themselves against antimicrobials by inducing stress responses. Therefore, higher γ-PGA concentrations (>2.0%) could exhibit better antibacterial efficacy, particularly against *S. aureus* and *S. dysenteriae strains* (Ajayeoba et al., 2019). Inbaraj et al. (2011) have reported an antibacterial mechanism of γ-PGA. They suggested that bacteria use non-specific (hydrophobic, electrostatic, and Vander Waals forces) and specific interactions between material surfaces and bacterial cell-membrane receptors for adhesion and proliferation. Moreover, bacterial cell growth could be inhibited by using anionic (bacterial cells have a negative surface charge) and hydrophilic materials. Therefore, the anionic and hydrophilic nature of γ-PGA might contribute to its antibacterial properties. Nevertheless, the antibacterial mechanism of γ-PGA should be further investigated. Recently Yu et al. (2023) investigated the antibacterial activity of four molar masses of γ-PGA against *Escherichia coli, Bacillus subtilis*, and yeast and they reported that the mechanism of action against tested microorganisms depends on the molar masses of γ-PGA and the microbial membrane structure. PGA of 2,000 kDa molar mass damaged the microbial cellular structure, resulting in the excretion of alkaline phosphatase, but PGA of 1.5 kDa molar mass affected the membrane permeability and the amount of soluble sugar. As the molar mass of γ-PGA increased, the intimicrobial activity increased. They found when the molar mass of γ-PGA was greater than 700 kDa, the MIC *for Escherichia coli* and *Bacillus subtilis* was less than 2.5 mg/mL. Chen et al. (2023) developed hydrogel composed from carboxymethyl chitosan (CMCS)/oxidized dextran (OD)/poly-γ-glutamic acid (γ-PGA) (COP) as antimicrobial and hemostasis of diffuse wounds. More interestingly, γ-PGA enabled the COP hydrogel to effectively absorb water and enrich platelets in the blood, which promoted the release of thrombin and coagulation factors and ultimately shortened the blood clotting time. In the COP hydrogel, γ-PGA was able to drain the surface moisture of the wound to enhance the surface adhesion. Moreover, γ-PGA could concentrate blood by absorbing plasma, and CMCS could electrostatically adsorb the negative red blood cells (RBCs). The antibacterial properties of CMCS and OD endowed the COP hydrogel with certain antibacterial effects. *In vivo* studies showed that the COP hydrogel significantly inhibited bacterial growth and promoted wound healing. Notably, the COP hydrogel exhibited broad-spectrum *in vitro* antibacterial activity against *S. aureus* and *E. coli* in both the zone of inhibition test and the liquid antibacterial test. In the rat tail diffuse hemorrhage wound model, the COP hydrogel showed superior hemostasis ability. Furthermore, the *in vitro* cytocompatibility test and *in vivo* biocompatibility test demonstrated the biocompatibility of the COP hydrogel. Therefore, the multifunctional COP hydrogel is expected to find different applications in wound hemostasis and healing.

#### 8.1.4 γ-PGA applications as biomedical adhesives and glues

Biological adhesives are used for hemostasis, tissue adhesion, and sealing of fluid and air leaks during surgery. Air leakage is a major complication of chest and lung surgeries, which is traditionally stopped by stapling or sewing (Richard and Margaritis, 2003). The use of fibrin, a biological adhesive, has been reported for this purpose. Fibrinogen, thrombin, and fibrin are retrieved from human blood transfusion and are generally highly biocompatible, but they can also serve as a source of viral infection. Fibrin, a common surgical adhesive, has poor tissue adhesion. In this regard, chemical crosslinking of γ-PGA and gelatin has demonstrated promising surgical adhesion and hemostatic properties that could be a better alternative to fibrin glue. Animal studies have revealed slow degradation of these hydrogels inside the body without severe inflammatory response. Otani et al. (1999) reported rapid solidification of PGA and gelatin-based WSC-crosslinked hydrogel as compared to fibrin glue, and it sealed the lung air leak more effectively than the fibrin glue. A mixed hydrogel of γ-PGA and gelatin was prepared using a crosslinker (1-(3-dimethylaminopropyl)-3-(ethylcarbodiimide) hydrochloride (EDC)), which exhibited shorter gelation time and higher bonding strength. A PGA and porcine collagen-based biological adhesive was also found to be better than fibrin in sealing lung air leakage.

### 8.2 γ-PGA applications in cosmetics

Proper moisturization and nutrition are vital to the health and beauty of human skin and hair. Over-dryness caused by low humidity is often detrimental to the skin and hair conditions. In winter, low temperature and dry air especially cause the dryness of the skin and hair, deteriorating the skin health conditions and even hardening or damaging the epidermis and electrifying the hair. To prevent the dryness of the skin, hair, and nail, cosmetic products such as skin essence, hand and body lotions, bath soaps, skin and body creams, hair gels, hair shampoos and mousse, and many other personal care products often contain certain moisturizers to provide the necessary moisturizing conditions to the skin and hair, and also to protect and beautify the skin and hair (Ho et al., 2005). γ-PGA and its hydrogel are considered as super moisturizer(s) in cosmetic products and personal care products showed excellent water absorption capacity and long-lasting good water retention, form soft tender smooth thin film with a matrix structure for controlled release function for water and other nutrients in the water, improve the elasticity of the skin, reduce wrinkles by anti-radical activity in the aging process, beautify, improve the health conditions of skin by increasing the natural moisturing factors in the stratum corneum, and reduce transepidermal water loss from the epidermis (Ho et al., 2005). γ- PGA hydrogel is a natural polymer hydrogel, with good biocompatibility and minimal cytotoxicity, it also includes a large number of free carboxyl groups in γ-PGA, which helps the hydrogel absorb water. However, when the water content of γ-PGA increases, its mechanical capacity decreases, hence various materials are frequently mixed to produce composite hydrogels to compensate for its inadequacies (Cai et al., 2023). PGA as moisturizer can enhance the effectiveness of personal care products for better exfoliation, skincare, hair care, and wrinkle-removing properties. PGA chemistry allows its homogenous miscibility and stability in the ingredient matrix of facial creams (Ben-Zur and Goldman, 2007). PGA also forms an elastic, smooth, and self-moisturizing soft film on the skin. It acts as an excellent hydrophilic humectant to enhance the synthesis of natural moisturizing factors including urocanic acid, lactic acid, and pyrrolidone carboxylic acid as compared to soluble collagen and hyaluronic acid. γ-PGA also improves skin elasticity better than hyaluronic and collagen and nourishes the skin for a smooth desirable appearance (Ben-Zur and Goldman, 2007). PGA hydrogel microscopy depicts a multi-baglike structure that can absorb moisture 5,000 times more than its weight. Salt content and pH influence the water-retaining capability of PGA hydrogel, which enhances the moisturizing capability of skin or hair formulations through optimum pH adjustments and electrolyte addition. Moisture release is important in facial masks for skin hydration and wrinkle removal (Ben-Zur and Goldman, 2007). γ-PGA has successfully served as hyaluronidase inhibitor’s active ingredient where it inhibited hyaluronidase-based hyaluronic acid degradation to maintain skin’s elasticity (Sung et al., 2005a). The composition was tested on fifty female participants (30–50 years) where it successfully maintained skin elasticity through the inhibition of hyaluronidase activity and alleviated allergenic reactions by restricting inflammatory cells’ permeability (Sung et al., 2005a). Moreover, a safe formulation of γ-PGA and natural extract (green tea or *aloe vera*) has been investigated as well. The result revealed a rise in pyrrolidonecarboxylic acid on the skin epidermis without affecting the constancy and quantitative balance of this humectant. PGA is also known to improve hair strength to support the bleaching process by promoting moisture-retaining capability and the formation of a protective barrier, which dilutes the chemical interactions of colorings applied with hair protein (Ben-Zur and Goldman, 2007).

Hyaluronic acid (HA) is a natural polymer and one of the components of polysaccharide extracellular matrix (ECM). Although pure HA is rigid and degrades quickly, it has been widely utilized in various ocular surgeries, can hasten wound healing, and has vast prospects in the cosmetics industry. Notably, PGA’s biodegradability, high water-retaining capacity, and ability to create amide bonds make it a valuable component for polyamide-PGA, outperforming hyaluronic acid in terms of improved moisturizing properties (Li et al., 2014; Cai et al., 2023). Bian et al. (2016) reported that γ-PGA can improve the shear resistance of HA. HA/γ-PGA hydrogel has a rich porous structure. Therefore, this hydrogel is a good material to provide a potential therapeutic method for future medical applications. Recently, an injectable hydrogel was developed through the photopolymerization of methacrylate-functionalized HA and γ-PGA. This hydrogel exhibited superior load-bearing capacity and toughness compared to conventional hydrogels (Ma et al., 2018).

### 8.3 γ-PGA applications in food industries

Beneficial nutration effects of γ-PGA have been previously reported. In mice, high-fat diets containing 3% γ-PGA significantly increased the serum HDL-cholesterol and significantly decreased the serum triglycerides (TG) compared to levels observed in the mice fed a high-fat control diet. However, the inhibition of intestinal high-fat absorption is one of mechanisms that improved the lipid metabolism in the mice fed with high-fat diet (Lee et al., 2013). Park et al. (2011) studied the effect of diets containing high molecular weight γ-PGA on adiposity and lipid metabolism in rats, suggesting that dietary supplementation with high molecular weight γ-PGA may act as a hypocholesterolemic agent. γ-PGA can serve as a non-toxic, edible, antimicrobial, and strong water-holding antioxidant in food industries. Thus, it has the potential to replace existing functional food supplements. γ-PGA role as a stabilizer and texture enhancer could promote the quality of wheat gluten (WG), starch, and their products (Xie et al., 2020). γ-PGA can stabilize fermented foods (yogurts) by maintaining microorganism viability, texture, food flavor, and aroma during production, transportation, and selling (Yu et al., 2018). It has been shown that γ-PGA can act as a bitterness-masking agent to curb the bitter taste of quinine, amino acids, minerals, peptides, and caffeine. It is also used as a thickener in fruit juice beverages (Feng et al., 2015). The anti-freezing feature of γ-PGA helps its utility as a cryoprotectant to sustain probiotics viability during freeze-drying (Siaterlis et al., 2009). Low molecular weight (<20,000 Da) γ-PGA possesses better anti-freezing properties than glucose and thus more efficiently protects *Lactobacillus paracasei* compared to sorbitol, sucrose, and trehalose (Bhat et al., 2013; Zhan et al., 2018; Jang et al., 2019). Multiple studies have demonstrated that 10% γ-PGA can protect *Lactobacillus paracasei* more effectively than 10% sucrose and offers better protection of lactobacilli during freeze-drying in comparison to sorbitol and trehalose (Siaterlis et al., 2009; Lim et al., 2012). A study was performed regarding the γ-PGA effect on doughnut moisture loss and oil absorption during deep-fat frying. A γ-PGA concentration of 1 g (100 g dough)^−1^ was found to effectively reduce (five-fold) doughnuts’ oil uptake to 0.2 g (g dough)^−1^ compared to an oil uptake of 0.7 g (g dough)^−1^ in regular doughnuts. Overall, γ-PGA-added doughnuts had better taste and appearance than regular doughnuts. Therefore, γ-PGA can be used as a potent oil-reducing agent in deep-fat fried foods. The antioxidant mechanism of γ-PGA was also found to shield the gastrointestinal tract against redox imbalance (Lee et al., 2020). Furthermore, γ-PGA enhances Ca^2+^ bioavailability via effective intestinal absorption. A single dose of γ-PGA was noted to enhance intestinal absorption in post-menopausal women, and it particularly benefited individuals with lower basal absorptive capacity (Tanimoto et al., 2007). Hu et al. (2008) studied γ-PGA-based possible induction of protein crystallization in xylanase, lysozyme, and glucose isomerase. They investigated high-molecular-mass (Na + salt:16106 Da) and low molecular-mass (Na + salt: 26105–46,105 Da) γ-PGA and noted γ-PGA-induced protein precipitation. In recent studies, Tamura et al. (2020) reported that γ-PGA could reduce postprandial glucose rise and plasma glucose levels. In an *in vivo* emperiment, mice were fed with different levels of a γ-PGA-containing diet or control diet for 91 days to suppress the initial rise in blood glucose levels. They found that blood and plasma glucose levels at 15 min were significantly decreased in the PGA group compared to the control group, suggesting that γ-PGA may effectively prevent metabolic syndrome by lowering blood glucose levels.

### 8.4 γ-PGA applications as a soil conditioner in agriculture

γ-PGA has promising agricultural uses as a new environmentally benign fertilizer synergist to boost nutrient uptake by plants. However, its agricultural application is hampered by the low yield and high cost of the production compared to conventional materials, because the production of γ-PGA by fermentation often requires glutamate and other high-cost components as substrates. However, some low-cost feedstocks are urgently required to overcome the economic and long-term barriers to biotechnologically producing γ-PGA (Feng et al., 2017). γ-PGA is particularly beneficial for soil fertility due to strong water retention, water solubility, high cation exchange capacity, high biodegradability, and metal-chelating and elements preserving capability to avoid their loss by rinsing with drainage water. The role of γ-PGA as an environment-friendly fertilizer synergist has increased its importance in plant growth-related soil dynamics (Chen et al., 2005). γ-PGA can significantly promote potassium (K), nitrogen (N), and phosphorus (P) uptake to increase plant biomass. It strengthens nutrient uptake by promoting root activity and biomass. γ-PGA is known to affect nitrogen (N) and carbon (C) metabolism in plants, which is represented by reduced free amino acids, nitrates, and high soluble sugar content (Zhang et al., 2017). Factors such as CO_2_ emission and carbon (C) and soil nitrogen (N) loss by leaching can demonstrate the potential of γ-PGA in agriculture. Zhang et al. (2016) reported a γ-PGA-based reduction in soil N leaching loss at an application rate of ≥0.2 g kg^−1^ soil. γ-PGA exerts only minor effects on the leaching of dissolved organic carbon (DOC), but it can significantly enhance the loss of soil CO_2_ gas. Yin et al. (2018) have revealed a significant reduction in soil organic carbon (SOC ≈ 0.4 g kg^−1^) and total nitrogen (TN ≈ 0.025 g kg^−1^) after the addition of γ-PGA. Contrarily, high γ-PGA concentrations (0.4 and 0.8 g kg^−1^ soil) increased the soil TN without affecting SOC content (Zhang et al., 2016). Furthermore, γ-PGA could enhance root activity and biomass by improving water-soluble nutrients’ uptake, and exogenous γ-PGA application can protect seedlings against adverse conditions (Wang et al., 2008; Yin et al., 2018). Moreover, γ-PGA has also been applied under biotic or abiotic stress conditions as an antimicrobial and biocontrol agent (Lee et al., 2018). γ-PGA application as a biological chelating agent could effectively alleviate toxic heavy metals accumulation in farmlands and crops (Pang et al., 2018). Currently, agricultural applications of γ-PGA are limited, but its broad range of applications can be predicted in the future for reducing nutrient loss, improving crop production, promoting fertilizer utilization, and sustaining the ecological function of soil (Zhang et al., 2017).

### 8.5 γ-PGA applications in wastewater treatment

The biodegradable and non-toxic nature of γ-PGA presents it as an eco-friendly wastewater treatment option. γ-PGA (MW ~ 5.8–6.2 × 10^6^ Da) could perform better than most conventional wastewater flocculants in treatment plants, which operate downstream of food fermentation processing units (Inbaraj et al., 2006). Ben-Zur et al. (2007) revealed that γ-PGA (MW ~ 9.9 × 10^5^ Da) efficiently removed basic dyes (98%) from the aqueous solution at pH 1 and remained reusable. Thus, a γ-PGA-based biodegradable and non-toxic adsorption system offers an eco-friendly solution to the dye industry. Chang et al. (2013) investigated the efficacy of γ-PGA added super-paramagnetic iron oxide NPs (γ-PGA/Fe_3_O_4_ NPs) for the elimination of heavy metal ions (Ni^2+^, Cr^3+^, Pb^2+^, and Cu^2+^). γ-PGA /Fe_3_O_4_ NPs demonstrated excellent metal removal efficiency as compared to individual treatments of γ-PGA or Fe_3_O_4_ NPs indicating its higher potential in wastewater treatment. During a study, *B. subtilis* 2063-produced γ-PGA (5.86106 Da) exhibited significant flocculating activity and thus can replace conventional synthetic flocculants (high environmental persistence) for wastewater treatment (Bhunia et al., 2012). Bajaj and Singhal (2011a) examined the *B. subtilis* R 23-derived γ-PGA (6.26106 Da) and revealed its excellent flocculation capability in wastewater treatment plants. Bodnár et al. (2008) investigated a novel complex of biodegradable NPs, *B. licheniformis* 9945a-derived γ-PGA, and bivalent lead ion. The complex remained stable in aqueous media under neutral, mild, and low-alkaline conditions, and exhibited effective water treatment and heavy metal-binding potential. Poly-γ-glutamic acid flocculant (PGAF)-based flocculation–sedimentation treatment could effectively eliminate SP-stabilized cement particles via gravitational settling of the flocs (Yanagibashi et al., 2019). However, higher ionic strength reduces the removal efficiency of γ-PGA, which might be due to its shrinkage and could be overcome by high intensity and rapid mixing. Several flocculation–sedimentation experiments have established the relationship between ionic strength and PGAF amount. The results suggested a higher efficacy of PGAF for cement suspension purification through flocculation–sedimentation, which could be further enhanced with rapid mixing at lower ionic strength (Yanagibashi et al., 2019). There are several other potential applications of γ-PGA, which are continuously expanding.

## 9 Conclusions and future perspectives

Natural biopolymers are becoming more and more in demand worldwide and are poised to take the place of traditional petro-based polymers. γ-PGA is an amino acid biopolymer that has recently attracted attention due to its biocompatibility, non-immunogenicity, biodegradability, and non-toxic properties. Due to its unique properties, it has been applied in various applications in healthcare, pharmaceuticals, water treatment, foods, and other important applications. However, it is expected that in the next five years, more research will be focused on the thorough evaluation of its biodistrubution, toxicity, and pharmacokinetics before using PGA-based materials in clinical trials for cancer therapy. γ-PGA is not susceptible to proteases, so it could provide better sustained delivery of conjugated drugs in the body. This review briefly covers γ-PGA-producing microorganisms, biosynthesis mechanism, limitations of γ-PGA production and the strategies for commercialization. Also, included the factors affecting γ-PGA production, downstream processing, characterization and identification approach and applications of γ-PGA. However, the main economic problem that prevents the commercialization of biopolymers is the price when compared to conventional counterparts. γ-PGA is one of the most expensive biopolymers with diverse applications. Even though there are potent γ-PGA-producing strains available, the cost of production and recovery are still high. Therefore, the screening for potential γ-PGA producer strains using a low-cost fermentation medium is the most upcoming need to reduce the overall production cost. To achieve this, more research exploring various types of agricultural and food industries wastes containing organic acids and amino acids that could be used directly as the fermentation substrate without supplementation with additional nutrients is needed. Also, whey permeates, a by-product of cheese processing in the dairy industry, also has attracted considerable attention due to its high nutritional content and functional properties. In addition, finding solutions for cost-effective γ-PGA production may also be possible through statistical analysis of large-scale γ-PGA production. γ-PGA has found extensive use in the medical field, particularly in the realm of continuous medication administration. One of the most promising attractive and perspective strategies for a large scale production is manipulating transgenic plants such sugar beat (*Beta vulgaris*) and cassava (*Manihot esculenta*) for producing polyglutamic acid by introducing a nucleic acid encoding a polyglutamic acid synthase A (pgsA), a nucleic acid encoding a polyglutamic acid synthase B (pgsB), and a nucleic acid encoding a polyglutamic acid synthase C (pgsC) into the plant. As a result, production costs will be significantly reduced for further commercialization. Finally, it will be expected that this attractive biopolymer could facilitate the development of more useful multifunctional biomaterials.

## Author contributions

KE: Conceptualization, Supervision, Writing – original draft. FA: Visualization, Writing – original draft. LN: Data curation, Visualization, Writing – review & editing. HA: Writing – review & editing, Data curation.
